# Advances, opportunities, and challenges in methods for interrogating the structure activity relationships of natural products

**DOI:** 10.1039/d4np00009a

**Published:** 2024-06-24

**Authors:** Christine Mae F. Ancajas, Abiodun S. Oyedele, Caitlin M. Butt, Allison S. Walker

**Affiliations:** a Department of Chemistry, Vanderbilt University Nashville TN USA allison.s.walker@vanderbilt.edu; b Department of Biological Sciences, Vanderbilt University Nashville TN USA; c Department of Pathology, Microbiology, and Immunology, Vanderbilt University Medical Center Nashville TN USA

## Abstract

Time span in literature: 1985-early 2024

Natural products play a key role in drug discovery, both as a direct source of drugs and as a starting point for the development of synthetic compounds. Most natural products are not suitable to be used as drugs without further modification due to insufficient activity or poor pharmacokinetic properties. Choosing what modifications to make requires an understanding of the compound's structure–activity relationships. Use of structure–activity relationships is commonplace and essential in medicinal chemistry campaigns applied to human-designed synthetic compounds. Structure–activity relationships have also been used to improve the properties of natural products, but several challenges still limit these efforts. Here, we review methods for studying the structure–activity relationships of natural products and their limitations. Specifically, we will discuss how synthesis, including total synthesis, late-stage derivatization, chemoenzymatic synthetic pathways, and engineering and genome mining of biosynthetic pathways can be used to produce natural product analogs and discuss the challenges of each of these approaches. Finally, we will discuss computational methods including machine learning methods for analyzing the relationship between biosynthetic genes and product activity, computer aided drug design techniques, and interpretable artificial intelligence approaches towards elucidating structure–activity relationships from models trained to predict bioactivity from chemical structure. Our focus will be on these latter topics as their applications for natural products have not been extensively reviewed. We suggest that these methods are all complementary to each other, and that only collaborative efforts using a combination of these techniques will result in a full understanding of the structure–activity relationships of natural products.

## Abstract 2

This review highlights methods for studying structure activity relationships of natural products and proposes that these methods are complementary and could be used to build an iterative computational-experimental workflow.

## Graphical abstract

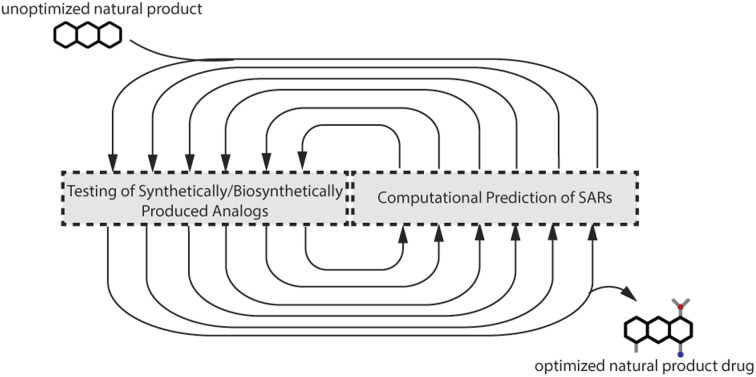

## 1. Introduction

Natural products (NPs) play an essential role in drug discovery – they have been used as medicines dating far back in human history, from before humans even understood the nature of chemical matter.^1^ In the modern era, NPs make up a large portion of the FDA-approved drugs with NPs and botanical mixtures accounting for 4.6% and NP derivatives accounting for an additional 18.9% of FDA approved drugs between 1981 and 2019.^2^ One potential explanation for the great utility of NPs in drug discovery is that they have evolved to target specific proteins and can therefore be used as drugs acting against those targets or their homologs. However, it is important to note that just because an NP has evolved to target a specific protein, does not mean that it is the ideal compound to treat a related disease. Many NPs are proposed to serve a defensive function for their producer by killing or inhibiting the growth of competitors. These compounds can be used against human pathogens or tumors that share the molecular target of that competitor. However, it is unlikely that these homologous targets will have identical binding site structures and therefore the NP may not function with as high an efficacy as it does against its natural target. In addition, there is likely little or no selection on NPs for other qualities that are necessary for making a successful drug, for example pharmacokinetic properties such as bioavailability in humans. This is because most NPs originate from environments quite different from the human body, for example soil or ocean environments or in plants. As a result, synthetic derivatives of NPs are generally more likely to be approved as drugs than NPs themselves.^3,4^ Another problem when using NPs against infectious agents or cancer is that the target cells can evolve resistance against the NP, rendering it ineffective at treating the disease.^5,6^

Because of these limitations, NPs must often be modified in a way that maintains or improves their activity while improving their pharmacokinetic properties in order to be used as a successful drug. In order to accomplish this, it is important to understand the structure–activity relationships (SAR) of the NP. SARs are a description of how a molecule's structure relates to its activity. A related concept is quantitative SAR (QSAR), in which mathematical models are used to quantitatively relate structure to activity. SARs are commonly used in medicinal chemistry to guide optimization of a lead compound.^7^ While there are many examples of SAR being used to develop NP leads into drugs (for example, caspofungin is a semi-synthetic analog of a natural echinocandin with lower toxicity^8^ and many rapamycin analogs with improved therapeutic properties have been developed^9^), SAR efforts are generally much more extensive for human-designed compounds.^10^ This is because synthetic compounds are generally less structurally complex and more amenable to synthetic diversification. Here we discuss experimental and computational methods that enable the study of SAR, their challenges and limitations, and propose how these methods can be applied to NP drug discovery. Our focus in this review is primarily on the methodology used for SAR studies, rather than the SARs of individual NPs. In addition, because experimental methods for SAR studies have been reviewed relatively recently,^11–14^ we will focus more on computational methods which have not been reviewed extensively in the context of NPs.

The most definitive way to determine how a functional group on a molecule contributes to its activity is to remove or chemically modify the group and measure the relative change in activity. To accomplish this, that analog must be obtained. For synthetic compounds, this would be accomplished through chemical synthesis. The same strategy can be applied to NPs. NP derivatives can be accessed through total synthesis, the complete chemical synthesis of the product from simple and commercially available precursors, or through synthetic derivatization. Due to the structural complexity of NPs, this process is more challenging than for compounds of synthetic origin, and we will discuss several total synthesis and derivatization strategies that have been developed to handle these challenges. The natural origin of NPs enables use of enzymes and even entire biosynthetic pathways to aid in their production, and we will also highlight synthetic studies that made use of natural or engineered enzymes to produce NP derivatives as well as those that engineered the entire biosynthetic gene cluster (BGC) of an NP to produce analogs. Another advantage of the natural origin of NPs is that it is likely that evolution has already sampled the chemical space around NPs, and those that are adaptive, perhaps to a different homolog of an ancestral target, will be selected for. Therefore, it is likely that there are evolutionarily-related BGCs which can be mined for analogs with a spectrum of activity against different targets.

**Fig. 1:**
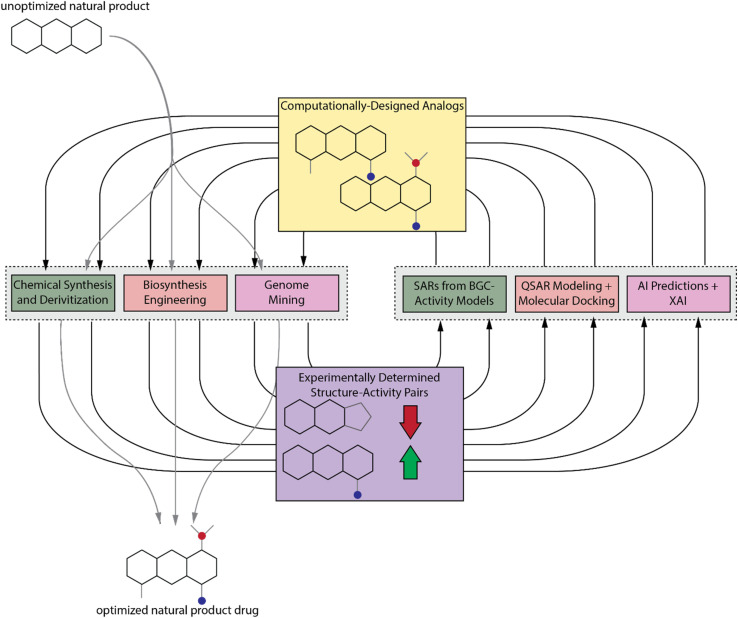
Proposed experimental–computational feedback loop. We propose that a combination of current experimental and computational techniques for studying SAR is necessary to fully understand the SARs of NPs. In this loop, experimental methods will be used to provide NP analog–activity pairs for training and validation of QSAR and XAI models and these models can in turn be used to guide synthetic and discovery efforts.

Despite the number of tools available to chemists for accessing NP analogs, it is still an extremely time-consuming process, and there may be some analogs that are inaccessible without considerable effort or development of new synthetic technologies. We propose that traditional computational drug design methods as well as more modern artificial intelligence (AI) methods, which are more commonly applied to synthetic compounds, can also be used to learn more about NP SARs. These computational results can then guide NP analog synthesis and discovery efforts by prioritizing those analogs that are more likely to improve activity (Fig. 1). We will also discuss these methods and provide suggestions for their application to NPs. First, we will discuss some recently reported methods for predicting bioactivity from BGC sequence and how those methods can be used to deduce SARs. We will then discuss traditional computer aided drug design (CADD) methods and AI techniques, with a focus on how explainable AI (XAI) can be used to elucidate SARs. The experimental and computational techniques are complementary. We propose that the best way to study NP SARs is with an experimental–computational feedback loop (Fig. 1). Due to the range of expertise needed for the different experimental and computational techniques, this approach will require that groups of interdisciplinary scientists collaborate to elucidate NP SARs and fully realize the potential of NPs in drug discovery.

## 2. Synthetic and semisynthetic approaches to SAR studies

### 2.1 Total synthesis for production of analogs

There have been many impressive total syntheses of NPs, which are often incredibly complex and therefore challenging to synthesize efficiently. In this review, we will only focus on a few selected examples where the same synthetic strategy was used to generate a large amount of chemical diversity, which could in turn be used for SAR studies. This discussion is not meant to be a comprehensive account of all studies that used total synthesis to study NP SARs or produce NP analogs but rather a general discussion of techniques and a highlight of a few studies that illustrate the use of total synthesis in NP SAR studies well.

**Fig. 2:**
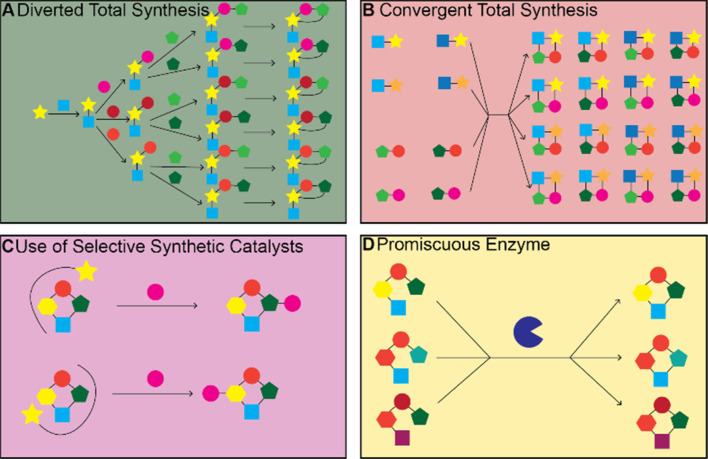
Synthetic strategy for diversification. (A) Diverted total synthesis and (B) convergent total syntheses are both total synthetic routes that diversify NPs; diverted synthesis has branch points while convergent synthesis feeds different starting materials or intermediates into the same downstream pathway. (C) Organo- and organometallic catalysts that interact with a substrate in a specific orientation can lead to site specific modification. (D) Promiscuous enzymes can act on multiple substrates to produce a variety of products.

**Fig. 3:**
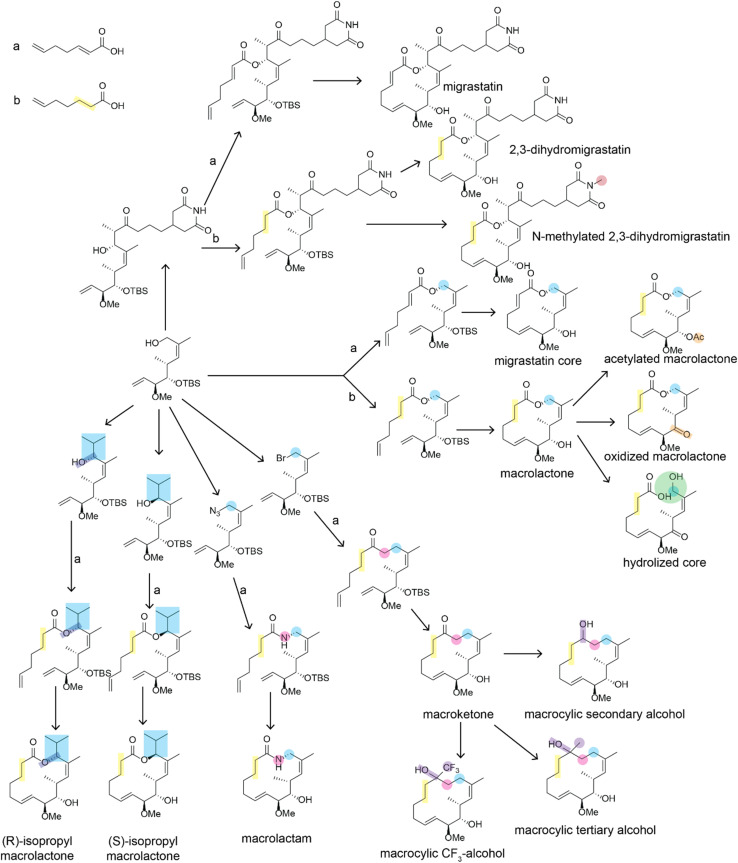
Divergent synthesis of migrastatin analogs described in ref. 18. Positions that are altered relative to the natural migrastatin are highlighted. For simplicity, reaction conditions are not shown. Arrows indicated with an a or a b indicate that two alternate reagents could be used at that step which introduce differences in the bond order of the bond between carbons 2 and 3. Bioactivity data on these structures is available in Table 1.

Most early total syntheses of NPs used a target-oriented approach, where the synthesis was designed to generate the single target compound.^13^ Analogs were difficult to access with this approach, as any modifications either had to be made at the end of the synthetic route or by making changes to intermediate steps while remaining compatible with the rest of the synthetic route. It is possible to design synthetic routes for specific analogs of interest, but this is inefficient to do on a large scale. The focus on target-based approaches began to change once the community recognized the importance of screening synthetic analogs of NPs to optimize them for therapeutic application^15^ and with the development of diversity-oriented synthesis approaches for small molecule library generation.^16,17^ One of the main approaches for accessing synthetic analogs of NPs for SAR studies is diverted total synthesis (Fig. 2A), a term first introduced by Danishefsky and applied to the synthesis of migrastatin analogs, resulting in some analogs with improved antitumor activity without sacrificing plasma stability (Fig. 3, and Table 1).^18^ This strategy, also referred to as collective total synthesis,^19^ involves first determining points on the target for diversification and then identifying the corresponding branch points from a common intermediate. It enables access to changes to the core of the molecule that cannot easily be installed at the end of the synthesis or by semisynthesis.^20^ Earlier branch points can lead to greater diversity, but also require more reactions to achieve.^15^ This strategy can result in modifications to the skeletal structure of the product, for example as is seen in the synthesis of pleuromutilin analogs by the Herzon group.^21^ In some cases, a single divergent strategy can be developed for a specific NP class. For example, the Baran lab developed a two-phase synthesis of terpenes inspired by the biosynthesis of terpenes, where the terpene skeleton is first built through cyclization and subsequently divergently oxidized.^14,22–25^

**Table 1:** Activities of migrastatin analogs reported in ref. 18

Compound name	4T1 tumor cell migration (IC_50_)	Stability (*t*_1/2_, mouse plasma)
Migrastatin	29 μM	>60 min
2,3-Dihydromigrastatin	10 μM	>60 min
*N*-methyl 2,3-dihydromigrastatin	7.0 μM	>60 min
Migrastatin core	22 nM	20 min
Macrolactone	24 nM	<5 min
Acetylated macrolactone	192 nM	NA
Oxidized macrolactone	223 nM	NA
Hydrolized core	378 nM	NA
Macrolactam	255 nM	>60 min
Macroketone	100 nM	>60 min
(*S*)-isopropyl macrolactone	227 μM	>60 min
(*R*)-isopropyl macrolactone	146 μM	>60 min
Macrocyclic secondary alcohol	8.9 μM	NA
Macrocyclic tertiary alcohol	3.1 μM	NA
Macrocyclic CF_3_-alcohol	101 nM	NA

Convergent synthesis is another strategy for generating diversity and involves feeding alternate starting materials or intermediates into the same downstream synthetic route, enabling diversification of structural motifs that must be installed earlier in the synthetic route (Fig. 2B). This approach has been applied to generate more than 300 macrolide antibiotic candidates.^26^ Another strategy for studying SAR relationships is pharmacophore-directed retrosynthesis.^13,27^ This strategy is similar to the truncated synthesis strategy^28^ in that it does not aim to synthesize the entire NP, but rather targets the pharmacophore necessary for activity from the outset of the total synthesis effort. Another similar strategy developed by the Shenvi group is to use computation to identify parts of the molecule that are important for target affinity and exclude unimportant but difficult to synthesize parts of the molecule. In one study by the Shenvi group, they aimed to improve the potency of salvinorin A, which has two epimers, one of which is significantly less active. They used computation to identify a change, in this case removal of a methyl group, that could be made to the molecule to favor the active epimer and improve ease of synthesis and used molecular docking to confirm the altered compound was likely to bind in the same pose; synthesis of the altered compound then confirmed the computational results.^29^ This type of analysis could also be used to first computationally confirm binding of a minimal molecule composed of just the proposed pharmacophore and then synthesizing it to confirm pharmacophore identity. There are a number of reviews that go into more depth on these general synthetic strategies with examples of successful applications and readers should refer to these reviews to learn more.^3,11–13,15,30–33^

One challenge of the diverted and convergent approaches is that many reactions must be carried out to generate the diverse products. The reactions needed increase exponentially with the number of branchings in the pathway and linearly with the number of parallel steps. Therefore, pathways that can be automated are ideal for producing a large number of analogs for SAR studies. Solid phase reactions, and in particular solid phase peptide synthesis, is especially amenable to automation, and peptide synthesizer machines are now commonplace. Solid phase peptide synthesis has been used for SAR studies of a number of important peptides including teixobactin,^34–46^ polymyxin,^47^ lysocin,^48^ jasplakinolide,^49^ daptomycin.^50–55^ However, there are still challenges with peptide solid phase synthesis. Some nonribosomal peptides contain rare amino acids that are not trivial to synthesize, and if a synthesis is not developed for these rare amino acids, they must be substituted in all synthetic analogs. Synthesis of these peptides also often requires multiple orthogonal protecting groups,^56^ and peptides with complex topology introduced by cyclizations cannot be easily synthesized by solid phase synthesis. Solid phase synthesis has also been used to synthesize polyketides, for example epothilone.^57,58^ While most automated syntheses of NPs are currently limited to those accomplished by a peptide synthesizer, there is currently substantial interest in developing general automated chemical synthesis platforms which could ultimately be used to generate larger diversity of NPs.^59–62^ Automated synthesis will also likely be complemented by future developments in computer-aided retrosynthetic planning, which can further automate the process of NP analog production.^63–67^

### 2.2 Synthetic modification of natural products for SAR studies

The total syntheses of NPs discussed above often require many steps and are not feasible for producing large quantities of different analogs. If a NP or biosynthetic intermediate can be isolated in large quantities from a native or heterologous producer through fermentation, then modification of the NP through chemical reactions becomes a valid strategy for accessing analogs for SAR studies. This approach is termed semisynthesis. The same methods can also be applied to the NP obtained through total synthesis rather than fermentation and is also referred to as late-stage functionalization. There are also already many existing reviews that cover this strategy^68–75^ so again we will limit our discussion to examples that illustrate general techniques and challenges involved in this approach.

Even if sufficient quantities of an NP can be isolated for input into functionalization reactions, there remain a number of challenges with this approach. These challenges mainly center around developing reactions with sufficient chemo-, site-, and stereoselectivity to modify the NP in the desired manner. NPs often contain multiple of the same reactive groups and therefore developing a reaction to target just one of them is challenging. There are often differences in reactivity for different instances of the same functional group due to differences in their local environment. If these differences are large enough, it becomes possible to modify the most reactive group selectively. Steric effects can also control which group is modified. If the reactivities are too similar or if the target for modification is not the most reactive group, then a catalyst that alters the relative reactivities of the different functional group in order to give the desired modification is required^74^ (Fig. 2C). In this section, we will highlight studies that demonstrated they could achieve selective modification at different sites on the same NP through alterations to the catalyst or reactants, rather than those studies that simply modified the most reactive or sterically accessible sites on a NP or those that relied on the incorporation of directing or protecting groups.

**Fig. 4:**
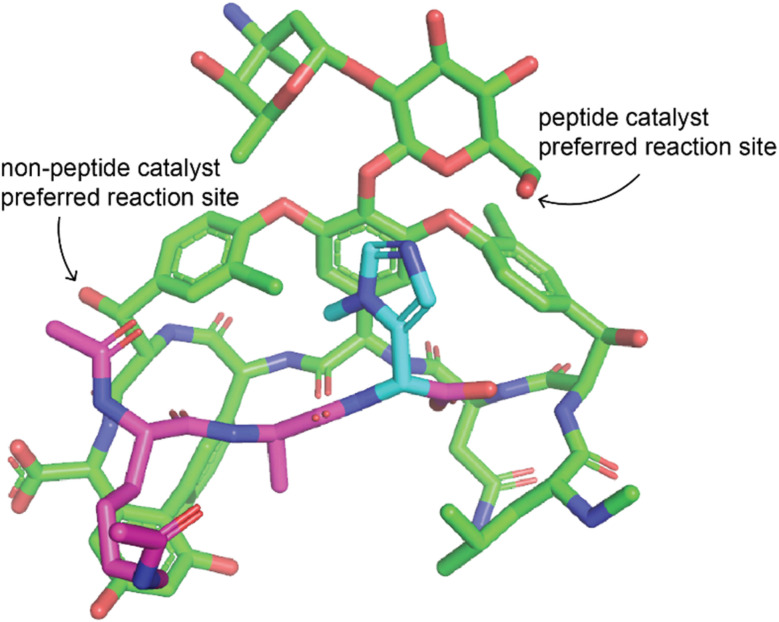
Example of peptide catalyst altering site selectivity of reaction. Peptide catalyst reported by the Miller lab that alters the site-selectivity of a thiocarbonylation reaction with vancomycin as a substrate.^79^ The peptide is a modified version of the target of vancomycin such that the catalytic residue is positioned near the desired modification site. Vancomycin is shown in green, the peptide in purple, and the catalytic residue of the peptide in cyan. Only the change to introduce the catalytic residue is shown, other changes made to the peptide are not shown. The structure was modified from PDB ID 1FVM with changes made manually, therefore this structure may not represent the actual structure and some dihedral angles may be inaccurate.

One very effective strategy pioneered by the Miller group is the use of peptide catalysts for site-selective modification. They have applied this strategy for acylation of hydroxyl groups of erythromycin^76,77^ and apoptolidin A,^78^ thiocarbonylation, deoxygenation, or lipidation of vancomycin,^79,80^ phosphorylation of teicoplanin hydroxyl groups,^81^ bromination of the aryl groups of vancomycin^82^ and teicoplanin.^83^ Some of the peptides used as catalysts for the modification of the glycopeptide antibiotics mimicked their natural target, D-Ala-D-Ala, to promote specific binding of the catalyst to the substrate (Fig. 4).^80–83^ Peptides are an ideal catalyst for this application because they are easy to synthesize and screen in order to identify catalysts that promote derivatization in different locations.^74,84^ In addition to peptides, other organocatalysts have also been used to selectively modify different positions in an NP. Chiral 4-pyrrolidinopyridine catalysts have been used to catalyze site-selective acylations of avermectin B2a and changes in solvent were shown to reverse the site-selectivity of the catalyst.^85^ Other examples include the use of bi(2-naphthol)-derived (BINOL) chiral phosphoric acids to alter site-selectivity of acylations of steroidal and flavonoid NPs.^86^

Organometallic catalysts have also been extensively applied in NP total synthesis and derivatization. Organometallic catalysts are especially useful in derivatizing NPs at C–H bonds, as the C–H bond is relatively inert and therefore difficult to activate for modification. C–H activation is a major area of research in chemistry and some of the resulting techniques have been applied to derivatization of NPs. The White group developed iron catalysts that they applied to oxidize C–H bonds in the NPs (+)-artemisinin^87^ and cycloheximide.^88^ They demonstrated that alteration of the catalyst's ligands can lead to catalyst-controlled selectivity and selective reaction at alternative sites previously thought to be too similar in reactivity for selective modifications.^89^ The Costas group has also applied similar iron catalysts for the site-selective oxidation of C–H bonds in various NPs.^90^ Oxidation of C–H bonds makes additional downstream modification possible, including those that alter the underlying scaffold such as ring expansion.^91^ Overall, while there has been considerable progress in this area, additional progress in catalyst development is needed before it becomes possible to easily edit any site on an NP by late-stage functionalization.

Some of the derivatization methods discussed here can also be used to insert handles, for example for click chemistry reactions, that can later be used to transform the NP into a probe, a strategy used by the Romo group and previously reviewed by them.^73^ While these analogs are not directly useful for SAR studies, they can be used to discover the molecular target of the NP, which is useful for guiding future SAR studies. Probe handles can be incorporated into any site on the NP so long as it does not interfere with target binding. The probe can then be added to cells or lysate from the target organism. Probes with a biotin or other affinity tag that can be used for pull-downs can then be used to enrich proteins that bind the NP. Proteomics can then be used to measure the enrichment of these proteins. Those with the highest enrichment are the most likely targets of the NP.^92^ Proteomic strategies for target identification have been extensively reviewed, for both synthetic and natural compounds.^92–102^ Once the target is known, a crystal structure of the NP bound to its target can be obtained. Crystal structures enable rational design and structure-based computational design, lessening the potential number of analogs that need to be screened before one with improved activity is obtained.

### 2.3 Enzymatic modification of synthetic products for SAR studies

In this section, we will focus on the use of individual enzymes applied to make specific modifications to an NP. We will discuss the engineering of full biosynthetic pathways in the next section. Enzymes are generally much more selective than the organo- and organometallic catalysts discussed previously. This is a trade-off because, while enzymes often only catalyze the reaction at a specific location on a molecule in a highly stereoselective fashion, they generally have extremely narrow substrate scopes. Therefore, enzymes often must be engineered for the desired substrate. We will present a few illustrative examples of how enzymatic modification can be incorporated into synthetic routes to enable selective access to more diverse products. For general reviews on biocatalysis for NP modifications readers should refer to ref. 103–105.

**Fig. 5:**
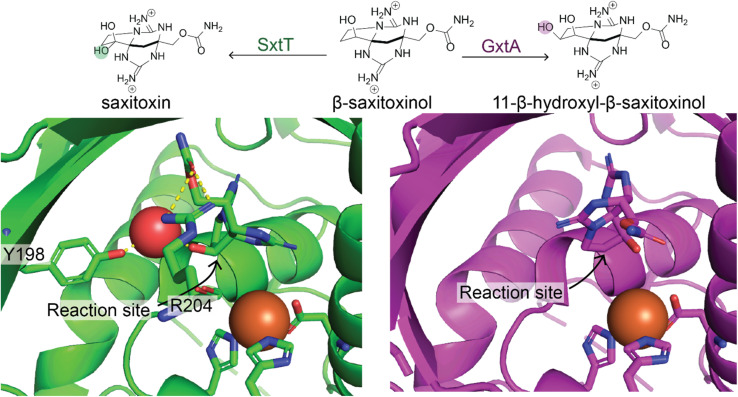
Natural enzymes with altered regioselectivity. Two natural enzymes, SxtT (green) and GxtA (purple) catalyze hydroxylation of β-saxitoxinol at two different sites. This is due to the different orientation of the substrate in the enzyme binding pocket. Residue R204 is involved in altering the orientation in SxtT relative to GxtA. Y198 is also positioned differently in SxtT enabling it to make a hydrogen bond with a water molecule that also interacts with the substrate.^107^

As is the case with the organic and organometallic catalysts, it is costly to develop an enzyme to catalyze a specific desired transformation. However, with sufficient effort, it is possible to engineer enzymes to act on novel substrates or even catalyze a different reaction. This was made possible by the Arnold group's pioneering work in directed evolution of enzymes, for which Frances Arnold won the Nobel Prize in 2018, in which large mutant libraries of an enzyme are screened to identify those that can catalyze the desired reaction. This process can be repeated multiple times starting from the best candidates from the previous rounds to lead to better selectivity and enzyme efficiency.^106^ Mutant libraries are often constructed by randomly mutating positions in the active sites of enzymes. Occasionally, naturally occurring enzymes provide a path for more rational engineering; for example, the SxtT and GxtA Rieske oxygenase enzymes have 88% sequence identity but install hydroxyl groups on different carbons in the saxitoxin scaffold. A study by the Bridwell-Rabb and Narayan labs compared structures of the enzymes to identify the positions important for determining site-selectivity and used this information to switch selectivity of the enzymes (Fig. 5).^107^

The Renata group has used natural enzymes from NP biosynthesis to access challenging precursors, simplifying the synthetic route and making it possible to invest more effort in producing analogs. Their efforts in this area include the use of natural enzymes for the hydroxylation of amino acids for production of cepafungin I analogs,^108^ GE81112 analogs,^109^ and oxidations of terpene scaffolds using P450s from terpene BGCs.^110^ In addition to natural enzymes, the Fasan and Renata groups have also used engineered enzymes for divergent NP chemoenzymatic synthesis, including the use of engineered P450s for C–H oxidation of terpenes or chiral terpene building blocks.^111–114^ This work is reviewed in more depth in ref. 115, and 116. Similar approaches have also been applied to the chemoenzymatic synthesis of polyketides. One study used synthetic intermediates, terminal PKS modules, and different combinations of glycosylases and P450s to produce a variety of structurally-related polyketides with different glycosylations and oxidation patterns.^117^ Mutations to P450s that catalyze multiple reactions in a cascade have been shown to alter regioselectivity – this strategy applied to a P450 from the tirandamycin BGC was used to generate five tirandamycin analogs.^118^

While enzymes are often applied to make specific modifications to a single substrate, another approach is to use a natural or engineered promiscuous enzyme on a library of compatible substrates to synthesize a variety of products (Fig. 2D). Enzymes with sufficient promiscuity can be used in convergent synthetic routes, where diverse intermediates are enzymatically transformed to produce diverse products.^119^ One example of this is the use of Stig cyclases and Fam prenyltransferases from hapalindoles and fischerindole BGCs, many of which were found to have a broad substrate tolerance, to produce 11 hapalindole derivatives and eight fischerindole derivatives.^120^ Another example of a promiscuous enzyme that can be used to generate many structural analogs is Ulm16, a penicillin binding protein (PBP)-like cyclase, which the Parkinson lab discovered to be highly promiscuous both in terms of precursor sequence and product ring size. They then used Ulm16 to generate libraries of cyclic hexa-, penta- and tetrapeptides from precursors produced by solid-phase peptide synthesis. This is especially notable for the tetrapeptides which are difficult to produce without the help of biocatalysis.^121^ This strategy could be applied to explore the chemical space around nonribosomal peptides and provide insight into their SARs.

A limitation of total synthesis, semisynthesis, and chemoenzymatic strategies for generating analogs is that a custom strategy must be developed for each NP of interest. For a total synthesis approach, a divergent or convergent route must be planned for each product class. For semisynthesis and chemoenzymatic late-stage derivatization, a catalyst must be chosen. If no catalyst exists that is both promiscuous and selective enough for general use, then a new catalyst must be designed for each desired modification site. This makes SAR studies by synthesis relatively low throughput. However, AI and automation is becoming more common in all areas of synthesis – for example in synthetic route planning,^122^ synthetic catalyst design,^123^ identification of synthetic steps that can be completed biocatalytically,^63^ and enzyme design.^124^ As these technologies become more advanced it should be possible to access more NP analogs for SAR studies.

## 3. Biosynthetic approaches to SAR studies

### 3.1 Natural product classes and nature's way of diversification

One method for producing derivatives of an NP is to edit or engineer the biosynthetic machinery that synthesizes it. To accomplish this, one must have an understanding of NP biosynthetic machinery; therefore, we will first introduce the biosynthesis of different NP classes to which this strategy has been applied.

**Fig. 6:**
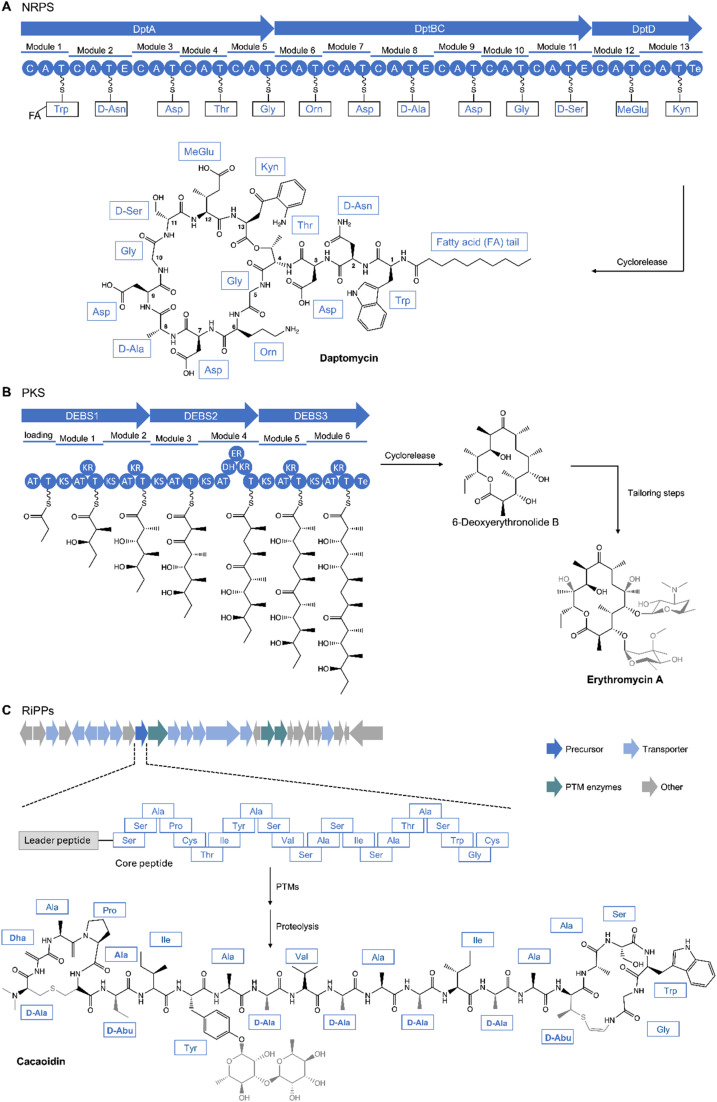
Schematic overview of natural product biosynthetic pathways. (A) Assembly-line logic of the biosynthetic routes for NRPS with their associated amino acid substrates to form daptomycin. (B) PKS modules with their starter and loading units to form erythromycin A, (C) mature RiPP cacaoidin formation.

Over time, advances in genomics and structural biology have unraveled the biosynthetic machineries and origins of NPs, offering insights into nature's diversification strategies. For instance, the pathway for non-ribosomal peptides (NRPs) are governed by NRP synthetases (NRPSs). NRPSs are composed of multi-modular enzymes following an assembly-line logic. Each adenylation (A) domain is dedicated to incorporating specific amino acids into the peptide chain. The activated building blocks are then transferred to the peptidyl carrier protein (PCP) or thiolation (T) domain while the condensation (C) domain catalyzes the peptide bond formation and the thioesterase (Te) domain releases the peptide chain (Fig. 6A).^125,126^ Similarly, a minimal set up of polyketide synthases (PKS), specifically Type 1 (T1PKS), consists of a module containing an acyltransferase (AT) domain to load an activated starter or extender unit such as acetyl-CoA, an acyl carrier protein (ACP), a ketosynthase (KS) domain for catalyzing a condensation reaction to extend the growing polyketide chain, and a Te domain which catalyzes the cleaving of the assembly line (Fig. 6B).^127,128^ Substrate specificity of the A and AT domains controls diversity of building blocks and starting units that make up the final product.^127,129^ Additional domains such as ketoreductase (KR), dehydratase (DH), enoylreductase (ER), methyltransferase (Mt) domains modify the polyketide core while NRPS have optional epimerization (E), *N*-methylation (NMt), heterocyclization (Cy), and oxidation (Ox) domains (Fig. 6).^126,130^ Another group of peptidic NPs are ribosomally synthesized and posttranslationally modified peptides (RiPPs).^125^ RiPPs are formed first by biosynthesis of a precursor peptide, comprising an *N*-terminal leader peptide and a C-terminal core region, by the ribosome. The leader peptide contains a recognition sequence which recruits post-translational modifying (PTM) enzymes to modify the core peptide, forming the mature peptide after removal of the leader peptide by peptidases; the modifying enzymes are tolerant of sequence diversity of the core peptide, providing a mechanism for diversification (Fig. 6C).^131,132^ More detailed information of the biosynthetic logic of these classes have been discussed in many recent reviews.^125,126,128,133,134^ Increasing understanding of the NRPS, PKS, and RiPP biosynthetic pathways, genetic manipulability, and enzyme promiscuity have made these important classes of NPs amenable to engineering, enabling production of analogs for SAR studies. Other classes of NPs such as terpenes have also shown amenability to engineering efforts.^135,136^

### 3.2 Methods to manipulate biosynthetic pathways and examples

**Fig. 7:**
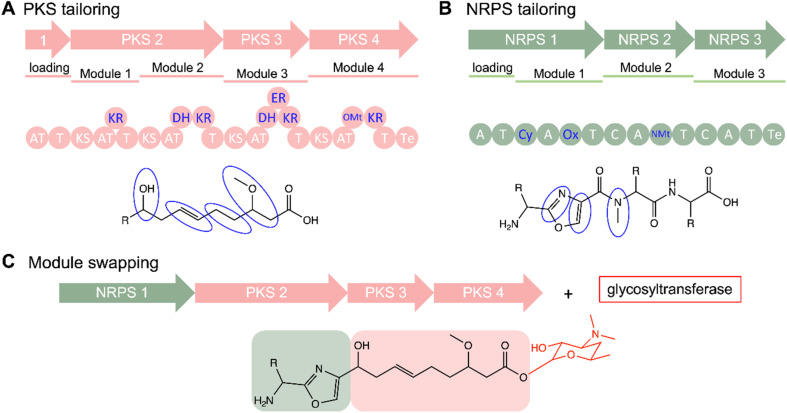
Expansion of PKs and NRPs NP diversity. (A) PKS and (B) NRPS systems feature multiple tailoring domains including noncanonical NMt, OMt, Cy, and Ox domains. (C) Stepwise assembly facilitates combinatorial biosynthesis such as module swapping. Additionally, independent tailoring enzymes like glycosyltransferases can add further modifications to the scaffolds during later stages as shown in the formation of a hypothetical glycosylated hybrid PK/NRP.

Combinatorial biosynthesis is a promising alternative to diversification of the NP arsenal, both structurally and functionally, taking advantage of genetic engineering techniques and the inherent properties of the biosynthetic pathways. These strategies play a crucial role in conducting studies on SARs, furnishing a versatile toolkit to probe the impact of structural variations on the biological activities of NPs. Combinatorial biosynthesis encompasses a spectrum of approaches, including domain/module shuffling, targeted mutagenesis, artificial pathways, directed evolution, manipulation of tailoring modifications (Fig. 7). Extensive reviews on these methods have been published in the past.^125,132,136–142^ Here, we highlight examples employing combinatorial biosynthetic approaches to create derivatives for SAR studies, along with related studies.

A shared property among most NPs like PKS and NRPSs that renders them highly amenable to combinatorial biosynthesis is their inherent assembly-line logic. This allows for predictable diversification by strategic deletion, insertion, duplication, and exchange of domains, modules, and units. According to the assembly-line logic, for example, deletion of modules relate to control of chain length, substitution of A or AT domains can alter building block incorporation while other changes can target stereochemistry and further tailoring steps.^143^ One of the pioneering examples applies the assembly-line logic of polyketides through transfer of genes involved in actinorhodin biosynthesis into the producers of medermycin/lactoquinomycin or dihydrogranaticin to produce mederrhodins A and B.^144,145^ Comparisons of the antimicrobial activity against a range of bacterial strains revealed that while mederrhodin A (lacking the OH group at C6) exhibited similar activity to medermycin against Gram-negative bacteria, it displayed reduced activity against Gram-positive bacteria. In contrast, mederrhodin B (lacking the cyclic lactone) was inactive against both types of bacteria,^145^ highlighting the importance of both the lactone and hydroxyl group in medermycin. The initial potential of combinatorial biosynthesis to generate products in a predictable manner spurred further interest for SAR studies. Given its role as the prototypical model of T1PKS and as the precursor for clinically relevant erythromycin and rapamycin, 6-deoxyerythronolide B synthase (DEBS) was used in hybridization studies. ATs in the DEBS pathway have been exchanged with those from other PKS clusters with different extender specificities including the rapamycin^146,147^ and avermectin PKS.^148,149^ In total, a large library of 61 6DEB analogs was systematically constructed,^150^ laying the foundation for further optimization of polyketide cores.^151–153^ Examples of such studies include those aimed at generating rapamycin analogs (rapalogues)^154,155^ and avermectin analogs for SAR studies at sufficient titers.^156^

In the context of NRPS, similar attempts have been made to use combinatorial biosynthesis for peptide analogs. One successful example of applying combinatorial biosynthesis to NRPS involves the NPs in the A21978 and A54145 complexes, including daptomycin.^157–159^ While these products are active against clinically relevant Gram-positive pathogens, only daptomycin has been developed as a clinical drug. Despite this, daptomycin's clinical use is limited due to inhibition through interaction with pulmonary surfactant, a mixture of compounds present in epithelial lining fluid in the lungs.^160,161^ Combinatorial biosynthesis has been used to produce analogs to probe the SAR of these related lipopeptides. This was accomplished through careful considerations of A, C, and T domain specificities^162^ to conduct gene deletions, exchanges, module shuffling,^126,141,163–165^ and lipidation, generating over 120 compounds; however, only around 40 were produced in sufficient amounts for further analysis. Effects of the modifications were analyzed against *Staphylococcus aureus* with and without 1% bovine surfactant (Fig. 6, and Table 2). The best results were from substitutions of Kyn13 to aliphatic Ile13 or Val13. Similarly, related A54145D and A54145E have relatively good antibacterial activities and arguably without surfactant inhibition.^166,167^ Further optimization was conducted by modifying eight positions of the core peptide.^162^ Notably, CB-182,390 had a minimum inhibitory concentration (MIC) of 2 μg mL^−1^ without surfactant and retained the same MIC with surfactant, indicating the importance of the modified positions Asn3, Asp9, and 3mGlu12, the latter of which has been shown to be correlated to antibacterial activity.^50^ These combinatorial engineering pursuits of the NRPS pathway of daptomycin and related peptides allowed for the interrogation of peptide core residues as well as preparation of non-proteinogenic amino acids such as 3mGlu. This has propelled further studies and derivatization of related lipopeptides by leveraging the importance of stereochemistry, Te domain cyclization, *N*-terminal modifications, and lipidations.^50,54,167–169^ Recently, the concept of evolution-guided identification of exchange units was developed for NRPS^170^ and *trans*-AT PKS engineering,^171^ greatly increasing the efficiency of engineering these biosynthetic pathways. The NRPS exchange unit strategy was applied to biosynthesize analogs of fellutamide B, a protease inhibitor, which resulted in a compound that is the best reported inhibitor of the *Mycobacterium tuberculosis* proteasome.^170^

**Table 2:** Daptomycin and lipopeptide antibiotics generated by combinatorial biosynthesis. Adapted from Baltz 2014.^172^ A schematic representation of Daptomycin with numbered amino acids is available in Fig. 6A

Compound	Amino acid at position									Side chain	*S. aureus* MIC (ug mL^−1^)		
	2	3	5	6	8	9	11	12	13		−Surf	+Surf	Ratio (±)
Daptomycin	D-Asn	Asp	Gly	Orn	D-Ala	Asp	D-Ser	3mGlu	Kyn	*N*-decanoyl	0.5	64	128
CB-182,107	D-Asn	Asp	Gly	Orn	D-Ala	Asp	D-Ser	3mGlu	Ile	Anteiso-undecanoyl	2	8	4
CB-182,106	D-Asn	Asp	Gly	Orn	D-Ala	Asp	D-Ser	3mGlu	Val	Anteiso-undecanoyl	4	8	2
A54145E	D-Glu	hAsn	Sar	Ala	D-Lys	moAsp	D-Asn	3mGlu	Ile	Anteiso-undecanoyl	1	32	32
A54145D	D-Glu	hAsn	Sar	Ala	D-Lys	moAsp	D-Asn	Glu	Ile	Anteiso-undecanoyl	2	4	2
CB-183,296	D-Glu	hAsn	Sar	Ala	D-Lys	moAsp	D-Asn	Glu	Kyn	Anteiso-undecanoyl	1	2	2
CB-182,390	D-Glu	Asn	Sar	Ala	D-Lys	Asp	D-Asn	3mGlu	Ile	Anteiso-undecanoyl	2	2	1
CB-182,561	D-Asn	Asp	Sar	Ala	D-Lys	moAsp	D-Asn	3mGlu	Ile	Anteiso-undecanoyl	1	2	2

While the previously mentioned examples were successful in generating a relatively substantial number of derivatives, the overall success rate of these approaches tends to be low as combinatorial editing of PKS and NRPS assembly are not as straightforward. Numerous recurring challenges are primarily due to disruptions in PSKS or NRPS systems which can be attributed to the impact of the gatekeeper domains and inter-domain communication.^128,139,173^ While recent efforts have reported the establishment of several high-throughput methods for NRPS and PKS engineering^149,174–178^ and the development of computational engineering tools such as ClusterCAD for multimodular T1PKS and NRPS,^179–181^ producing libraries of derivatives for SAR studies is limited by low production titers.^182,183^ Another challenge is that derivatives generated from manipulation of the assembly-line typically cover a limited chemical space, mainly modifying the reduction level and side chains of the core polyketide or peptide scaffold and may not necessarily exhibit improved activity compared to the parent compound. One example is from a recent SAR study on a hemiacetal-less rapamycin with diminished activity, suggesting nature has already optimized some of these scaffolds.^184^ Given the invaluable insights gained from SAR studies of NPs for their optimization for clinical use, alterations to these enzymes may necessitate significant modifications that may otherwise not yet be sampled by nature. This could include the incorporation of non-natural building blocks, a possibility achievable through combined approaches like metasynthesis.^185^

On the other hand, nature continues to provide examples that inspire other ways of diversification. Nature has ingeniously exploited the shared features between PKS and NRPS, as evident from multitude of hybrid NRPS-PKS NPs^186–189^ such as antitumor bleomycin^190^ and FK520,^191^ and vatiamides A–F.^192^ These examples showcase the versatility of hybrid PKS and NRPS assemblies and highlight the potential of hybrid combinatorial pathways for expanding biosynthetically-accessible chemical space. Recent examples exchanged domains from similar antimycin-like hybrid enzymes to generate novel neoantimycin and JBIR-06 derivatives with relatively productive yields.^193,194^ NRPS/PKS engineering was also used to produce a rapamycin analog. While the rapamycin core is biosynthesized mostly by PKS which have been extensively manipulated for production of other rapalogues, its gene cluster also has an NRPS gene that incorporates pipecolic acid but is promiscuous enough to accept alternative substrates such as l-proline, enabling production of additional analogs.^195^ This strategy has also been attempted in fungal hybrid system to swap non-cognate PKS and NRPS modules with mixed success.^196–198^ A better understanding of PKS and NRPS compatibility is imperative for hybrid engineering.

Despite the still limited number of RiPPs that have received clinical approval, RiPP enzyme engineering has emerged as a promising avenue to access peptides that might be better suited as drugs than their natural counterparts, as emphasized by recent reviews.^132,133,140,142^ In RiPP biosynthesis, the organization of the leader peptide, core peptide and PTM enzymes can be viewed as parallel to the modular logic of NRPS and PKS, and it facilitates even easier manipulation for peptide diversification compared to NRPS engineering. Moreover, since RiPPs are directly gene-encoded, precursor peptide mutants can be generated from mutagenesis and recombinant techniques, offering a facile approach to creating libraries of derivatives. One example is from the Müller lab on the promising RiPP antibiotic, darobactin, by heterologous expression to increase titers and identify analogs with improved activity.^199^ Technologies for generating RiPP analogs in high-throughput have been improving rapidly. A novel nanoFleming platform was used to screen for bioactive molecules, 11 of which had improved activity against *Enterococci* and *Staphylococci* strains.^200^ Another recent study generated a library of over 90 000 ubonodin lassopeptide variants.^201^ A select 15 of these variants showed antimicrobial activity against *Burkholderia cenocepacia* while one variant (H17G) had a lower MIC than the wild-type ubonodin, which already has a MIC comparable to clinically approved antibiotics.^201,202^ Moreover, the large data set allowed the generation of a deep learning model to predict RNAP inhibition which was also validated by RNAP inhibitory activity of the variants. Compared to NRPS and PKS engineering efforts, these SAR studies of RiPPs sample a large chemical space in sufficient titers for activity assays in a high-throughput manner. Moreover, the potential of RiPP engineering can be expanded to generate artificial libraries inspired by hybrid RiPP pathways and NRP mimics.^125,140,203,204^

Post-tailoring enzymes which catalyze reactions including glycosylation, halogenation, and alkylation, are commonly observed in many classes of NPs.^205^ These tailoring modifications decorate the scaffolds of NPs to increase the structural diversity and pharmaceutical applications, providing another catalytic toolbox to probe SAR. One versatile tailoring reaction is the addition of sugars by glycosyltransferases (GTs) which improves solubility and bioavailability. While this has been well-explored for polyketides such as in erythromycin^206,207^ and glycopeptides like mannopeptimycin,^208^ RiPPs are an interesting new target as only a few of these glycosylated RiPPs have been isolated such as cacaoidin,^209^ glycocins,^210^ and NAI-112.^211,212^ A few glycopeptide engineering tools have been developed and applied to produce peptides which showed inhibitory activity against *Bacillus cereus* with a lower MIC than sublancin, a natural glycocin.^213^ A high-throughput screening assay (SELECT-GLYCOCIN) was developed for facile generation of O- and S-linked glycopeptide (enterocin-like) libraries in which di-glycosylated variant G16E-H24L showed improved activity against *Listeria monocytogenes*.^214^ In combination with previous studies reporting relaxed substrate specificity of S-glycosyltransferases,^215^ these strategies provide powerful tools for production of novel glycopeptides. Apart from RiPP glycosylation, other recent reports of improved activity from combinatorial biosynthesis using tailoring enzymes across polyketides,^216,217^ NRPs,^218^ and other classes^219–222^ highlight the power of this strategy.

## 4. Genome mining of natural products: unveiling evolutionary relationships between biosynthetic gene clusters for valuable SAR insights

### 4.1 Evolution and SAR of natural products

NPs, also called secondary or specialized metabolites, are thought to help their producing organisms adapt to specific ecological niches or lifestyles.^223^ Therefore, the genes that are essential for producing NPs should be under selection when their product provides a fitness advantage to the organism. Changes in environment could lead to changes in selective pressures; for example, if a new competing organism enters an environment, there could be selective pressure for the original organism to produce compounds that inhibit the growth of the new competitor. Introduction of antimicrobial resistance genes into a population would also likely lead to a change in selective pressures on genes that produce antimicrobial compounds. One challenge facing this work is that the true ecological role of NPs is often unknown, and may not be the same as the potential clinical applications.^224^ Some have even suggested that NPs do not serve specific adaptive roles and are instead neutrally evolving offshoots of primary metabolism or a way to dispose of unneeded precursors.^225^ However, production of NPs is costly and there is mounting evidence that they are under selection. For example, there is evidence that BGCs that produce synergistic compounds coevolve, in the case of the β-lactams and β-lactamase inhibitors (such as clavulanic acid) or pairs of compounds that inhibit a target at different sites, such as the streptogramins.^226^ There is also evidence of convergent evolution of chemical structures, for example, dentigerumycin and gerumycin from the fungus-growing ant system, which have similar activities and chemical structures but unrelated BGCs.^224^ Convergent evolution is also observed among unrelated BGCs that produce similar β-lactam scaffolds.^227^ Another example of convergent evolution of BGCs are the multiple unrelated pathways for producing phosphonate NPs such as fosfomycin and fosmidomycin.^228,229^ Understanding the ecological roles of NPs and the mechanisms behind BGC evolution, specifically how selection acts on genetic variation to give rise to active compounds, can provide insight into NP SAR.

There are several existing research articles and reviews that investigate what is known about the evolutionary mechanisms and dynamics that give rise to diverse NP structures. Here, we will just highlight some of the common themes across these publications. Genetic variation in BGCs capable of leading to differences in product structure can arise through several mechanisms including *de novo* assembly, gene duplication, gene diversification, rearrangements, and horizontal gene transfer.^129,223,224,227,230^ Medema *et al.* performed a comprehensive analysis of BGC evolution and observed the same evolutionary mechanisms discussed in these reviews and frequent merging of smaller “sub-clusters”. These sub-clusters appear to function as independent evolutionary units, which can be transferred and recombined between different BGCs, giving rise to new chemical entities.^231^ It has also been proposed that enzymes from secondary metabolic pathways are more promiscuous than those from primary metabolism, enabling diversification and faster evolution.^223,230,232,233^ Changes to the structure can also occur through the gain or loss of tailoring enzymes, leading to different modifications of a shared scaffold.^129^ One cluster may also produce multiple compounds due to incomplete modification by a tailoring enzyme or perhaps differences in the expression of the tailoring enzyme relative to the core enzymes.^234^ Such clusters are a source of closely related compounds that could be used for SAR studies.

### 4.2 Bioinformatics tools and databases for gene cluster comparison for natural variant exploration

The advancement of scientific research has been propelled by the advent of cutting-edge technologies like genomic sequencing, curated databases, and bioinformatic tools powered by machine learning to facilitate the examination of gene clusters to uncover bioactive secondary metabolites.^235^ Software for analyzing and comparing sequences such as BLAST,^236^ Diamond,^237^ and HMMer^238^ enable exploration of large quantities of genetic data. A drawback of these methods is that they only annotate one gene at a time. Therefore, multiple BGC-specific tools have been built on these technologies to enable the characterization and comparison of multiple genes in order to identify and analyze BGCs. Some of the openly available BGC-computational tools include CLUSEAN,^239^ NP.searcher,^240^ antiSMASH,^241^ MultiGeneBlast,^242^ DeepBGC,^243^ RODEO,^244^ BiG-SCAPE,^245^ BiG-SLiCE,^246^ CORASON,^245^ EvoMining,^247^ PRISM,^248^ ARTS,^249^ ClusterScout,^250^ and the lately developed cblaster,^251^ clinker,^252^ CAGECAT,^253^ and lsaBGC.^254^ To understand how changes in BGCs that occur over evolutionary history influence the structure and activity of their products, it is necessary to compare evolutionarily related BGCs to identify the insertions, deletions, duplications, and recombinations that result in changes in product structure. Many BGC-computational tools provide methods by which to compare clusters (Table 3). AntiSMASH has knownclusterblast and clusterblast, which enable comparison of BGCs to characterized BGCs from the MIBiG database and BGCs from the larger antiSMASH database, respectively. These methods identify clusters that have homologous genes, as defined by a set threshold of sequence similarity, and provide a visual representation of which genes in the pairs of clusters are homologous and a percent similarity score.^255^ Knownclusterblast and clusterblast are limited by their reliance on specific databases for comparison and can only be used to analyze BGCs that belong to well-established biosynthetic classes that are identified by antiSMASH. Other tools, such as MultiGeneBlast, ClusterScout, lsaBGC, and cblaster enable searching for multiple genes, which the user specifies, that co-occur against the NCBI database using BLAST or HMMER searches.^242,250,252,254^ Clinker provides a mechanism for visualizing results from cblaster or other search methods, coloring genes by homology, and connecting homologous genes by paths shaded by the level of sequence identity. The cblaster-clinker workflow was recently combined into a single user-friendly webserver, CompArative Gene Cluster Analysis Toolbox (CAGECAT).^253^ RODEO uses a different approach, performing queries on a single gene family, but subsequently allows for the analysis of gene co-occurrence patterns in the genomic neighborhood of the query.^244^ The EvoMining approach also searches first for individual genes, specifically enzymes related to those from primary metabolism that may have functionally diverged to become part of secondary metabolism, and then analyzes the surrounding genome for similar domains that could indicate a BGC. This method enables the identification of previously unknown classes of BGC.^247^

**Table 3:** Methods for comparing BGCs. Query searches are searches that use one or more domains or genes from a cluster as a query, untargeted clustering compares all BGCs in an input database and does not rely on a specific query

Method	Type of search	Type of visualization
antiSMASH	Query	Colored by homology
MultiGeneBLAST	Query	Colored by homology
ClusterScout	Query	Colored by homology, BGC similarity network
Cblaster/clinker/CAGECAT	Query	Gene presence/absence table, colored by homology
lsaBGC	Untargeted clustering	Colored by homology, gcf phylogeny heatmap
RODEO	Query	Colored by homology
BiG-SCAPE	Untargeted clustering	BGC network, BGCs colored by matching domains
BiG-SLICE	Untargeted clustering	BGCs colored by matching domains
CORASON	BiG-SCAPE cluster	Colored by homology

All the methods discussed so far rely on the user identifying a specific BGC or family of BGCs to use as a query. However, to understand the broader evolutionary history of BGCs it will likely be necessary to identify multiple groups of related BGCs. There are several methods for clustering BGCs based on sequence similarity of their genes or shared biosynthetic domains. Biosynthetic Gene Similarity Clustering and Prospecting Engine (BiG-SCAPE) calculates the distance between clusters based on a combination of shared types of protein family (PFAM) domains, percentage of shared adjacent domains, and sequence identity which is measured using HMM profiles to improve computational speed. These scores are weighted differently for different classes of BGCs to account for class-specific evolutionary dynamics. These distances are then used to build similarity networks of BGCs to cluster them into gene cluster families (GCFs); different thresholds allow for hierarchical clustering.^245^ While BiG-SCAPE was designed to process many clusters quickly, it is not fast enough to process all putative BGC sequences in one run. Biosynthetic Genes Super-Linear Clustering Engine (BiG-SLiCE) was developed to address this issue and works by first converting BGCs into a vector representation of the absence/presence and similarity bitscores resulting from a gene search using profile HMMs (pHMMs). Then the BIRCH clustering algorithm, which runs with near linear complexity, is used to cluster large numbers of BGCs into GCFs.^246^ Both BiG-SCAPE and BiG-SLiCE offer interfaces that allow for the visualization of shared domains between members of the same GCF, allowing for the identification of evolutionarily conserved core biosynthetic proteins. BiG-SCAPE also allows for the visualization of the BGC similarity network. A complementary method to BiG-SLiCE, clust-*o*-matic, uses an all-versus-all distance matrix of BGCs based on sequence similarity and agglomerative hierarchical clustering; these two methods were found to generally agree with each other.^256^ LsaBGC also provides a method to cluster BGCs, focusing on genes identified as homologous using OrthoFinder2, rather than PFAM similarity, and a synteny score similar to that of BiG-SCAPE. Another advantage of lsaBGC is that it can calculate various evolutionary statistics, such as the rate of synonymous and nonsynonymous mutations for homologous genes, in addition to analyzing overall gene co-occurrence patterns, potentially revealing parts of biosynthetic enzymes that are under purifying or directional selection which could correlate with activity of the product.^254^

Finally, while all the methods we have described so far can identify potentially homologous genes between clusters, they do not provide insight into the evolutionary relationships of the clusters. One method for learning more about evolutionary relationships is to build phylogenetic trees for individual genes that are shared between the clusters of interest.^257^ This type of analysis can be especially useful when applied to genes whose evolutionary history is highly correlated with the product's structure, for example, *trans*-acyltransferase polyketide synthases (*trans*-AT PKS) ketosynthase (KS) domain.^258^ However, in many cases, these results are only applicable to the individual domains or proteins and not the whole cluster because frequent recombination events in BGCs mean that the evolutionary history of different proteins in the cluster may be distinct.^231^ CORe Analysis of Syntenic Orthologs (CORASON) can be used to generate multi-locus phylogeny of a set of related BGCs using the sequence of one or more genes conserved across the BGCs and uncovers all clades that may be accountable for the biosynthesis of a family of NPs. CORASON has also been integrated with BiG-SCAPE to build phylogenetic trees for GCFs identified by BiG-SCAPE.^245^

The successful application of the tools discussed above requires high-quality and open sequence databases. Available databases include BAGEL,^259^ antiSMASH-db,^260^ IMG-ABC,^250^ MIBiG,^261^ and BiG-FAM.^262^ BAGEL is a web-based database that provides sequences of putative bacteriocins and RiPPs.^259^ antiSMASH-db^260^ and Integrated Microbial Genomes Atlas of Biosynthetic gene Clusters (IMG-ABC)^250^ include both experimentally verified and predicted BGCs. BiG-FAM is a database of putative BGCs clustered into GCFs, enabling comparisons of related BGCs for users who cannot run clustering of BGCs themselves.^262^ These databases are useful for genome mining efforts and for evaluating sequence variation between related BGCs, which could provide insight into how they have evolved to have different structures and functions. MIBiG is unique in that it is curated to BGCs with experimental evidence linking them to a specific NP.^261^ MIBiG also interfaces with NPAtlas,^263^ a database of NP structures, enabling the study of BGC-structure–activity relationships.

### 4.3 Example case studies that illustrate how BGC comparison informs knowledge of SAR

Various combinations of the techniques and datasets described above have been used to successfully identify structurally related compounds produced by evolutionarily related BGCs. These types of linkages enable the understanding of how evolutionary processes shape NP structural diversity and alter their bioactivities, possibly for the purpose of adaptation. Several existing reviews describe the use of phylogenetic technologies in NP studies.^257,264–266^ Here, we will highlight some studies that applied these approaches to isolate compounds that were related to known compounds and compare the activity of these different structural analogs.

**Fig. 8:**
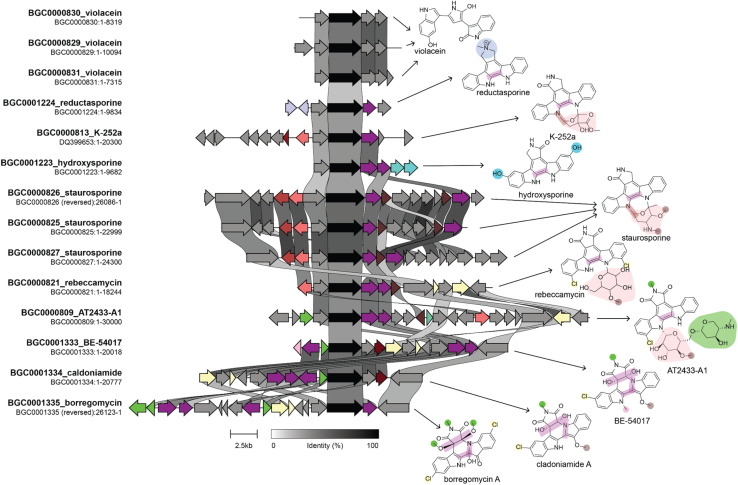
Genome mining for tryptophan dimer NP analogs. This figure shows a comparison of BGCs and their corresponding products. The BGC image was created using clinker with BGCs retrieved from the MIBiG database. The genes in black are homologs of *staD*, the gene used as a handle for eDNA genome mining by the Brady lab. Connections between genes indicate percent sequence identity. We identified several genes that lead to structural divergence and colored them based on enzymatic activity in a manner consistent with the coloring of functional groups on the product structure. Homologous genes are given the same color even if they have divergent enzymatic activities. Note that not all structural differences in the product are due to gene gain or loss. For example *staC* and *rebC*, which are involved in the conversion of chromopyrrolic acid to the staurosporine and rebeccamycin aglycone, respectively, result in different oxidation states at the C-7 position, which suggests that differences in these enzymes' catalytic activity results in structural divergence. Information on the biosynthesis of these compounds used in the figure was obtained from ref. 268–270, and 277–279.

The Brady lab developed a phylogenetic approach to identify BGCs that produce analogs of known compounds from eDNA libraries. First, their approach involves selectively amplifying core biosynthetic genes whose phylogeny is linked to product structure, sequencing the amplicons, and then building a phylogenetic tree from the resulting sequences and those from known BGCs. Finally, heterologous expression is used to isolate the product of interest. They performed this process using the chromopyrrolic acid synthase gene from tryptophan dimer BGCs to identify several novel tryptophan dimer NPs (Fig. 8, and Table 4), including those from previously unknown subclasses. These compounds all had different degrees of cytotoxic activity against tumor, fungal, and bacterial cells and likely have different molecular targets.^267–270^

**Table 4:** Bioactivities of tryptophan dimer NP analogs. This table shows activities of different tryptophan dimer analogs shown in Fig. 8. Abbreviations used in table: MIC = minimum inhibitory concentration, MTD = maximum tolerated dose, IC_50_ = half maximum inhibitory concentration^268–270,277–279^

NPs	Bioactivities				
	MIC in μg mL^−1^: *S. aureus*	MIC in μg mL^−1^: *E. Coli*		IC_50_ (μM): human HCT116	MIC in μg mL^−1^: *C. albicans*
Reductasporine	105	>150		503	36.3
Hydroxysporine	>150	>150		36.5	36.0
Erdasporine A	4.2	—		4.3	—
Erdasporine B	4.2	—		5.3	—
Erdasporine A	8.5	—		13	—
Violacein	6.2	>200		5–10	4.375
	MIC in μg mL^−1^: *S. aureus*	MIC in μg mL^−1^: *E. Coli*	MIC in μg mL^−1^: *S. faecalis*	IC_50_ (μM): human HCT116	IC_50_ (μM): P388 leukemia
Rebeccamycin	1	>125	8	0.41	6.0
	MIC in μg mL^−1^: *S. aureus*	MIC in μg mL^−1^: *E. Coli*		IC_50_ (μM): human HCT116	MIC in μg mL^−1^: *S. cerevisiae*
Arixanthomycins A	1.6	>50		0.15	>50
Arixanthomycins B	25	>50		5.14	>50
Arixanthomycins C	>50	>50		25.42	>50
	MIC in μg mL^−1^: *M. tuberculosis*	MIC in μg mL^−1^: *S. grisues*		IC_50_ (μM): PfPK5	IC_50_ (μM): PKnB
Staurosporine	5–50	125		1.0	0.60
	MIC in μg mL^−1^: *M. tuberculosis*	MIC in μg mL^−1^: *S. grisues*		IC_50_ (μM): PfCDPK1	IC_50_ (μM): PKnB
K252a	5–50	12.5		0.045	0.096
	MIC in μg mL^−1^: *S. aureus*	MIC in μg mL^−1^: *E. coli*		IC_50_ (μM): human HCT116	MIC in μg mL^−1^: *B. subtilis*
Borregomycin A	>25	>25		1.2	>25
Borregomycin B	0.20	>25		1.4	0.20
Borregomycin C	0.39	>25		1.9	1.6
Borregomycin D	3.1	>25		3.9	3.1
	MIC in μg mL^−1^: *S. pombe mutants*	MIC in μg mL^−1^: *E. coli*		IC_50_ (μM): human HCT116	MIC in μg mL^−1^: *B. subtilis*
BE-54017	0.031	—		0.079	—
Cladoniamide	0.078	—		0.0088	—
	MIC in μg mL^−1^: *S. aureus*	MIC in μg mL^−1^: *M. luteus*		MTD (mg kg^−1^): P388 leukemia	MIC in μg mL^−1^: *B. subtilis*
AT2433-A1	16	<0.25		2	4.0
AT2433-A2	16	1.0		—	8.0
AT2433-B1	32	0.25		4	8.0
AT2433-B2	32	4.0		—	64.0

The Brady lab has also applied this approach to KS_β_ domains from anthracycline and pentangular polyphenol BCGs. This resulted in the discovery of new anthracyclines, arimetamycins A–C, which were produced by a gene cluster most closely related to the steffimycin BGC. The cluster had additional glycosyltransferases, and the arimetamycins were glycosylated with additional sugars not previously found in the steffimycin family. Arimetamycin A, which was glycosylated with two rare sugar moieties, showed improved activity against multiple cancer cell lines, including two multidrug-resistant cell lines, compared to doxorubicin and daunorubicin. This indicates that these sugars could be important for improving activity and that the glycosyltransferases in the arimetamyicn BGC could help their host compete against microbes that had evolved resistance to monoglycosylated steffimycins.^271^ The same approach applied to the pentangular polyphenol family of polyketides resulted in the discovery of arixanthomycins A–C, which differ from previously discovered pentangular polyphenols in many ways, including the addition of a carboxylated oxazolidine ring, glycosylation at C-13, and different oxidation states of some of the rings. The arixanthomycins were found to have antiproliferative activity, with arixanthomycin A being the most active. The authors attributed the improved activity to the sugar moiety present on arixanthomycin A but not on arixanthomycin B and C.^272^

The glycopeptide antibiotics (GPAs) are an especially interesting class to study with a phylogenetics approach because resistance mechanisms do not provide equal protection against all GPAs,^273^ and some GPAs may have evolved to escape those mechanisms. Several studies have built phylogenies of different protein domains in GPA clusters to reveal the natural history of glycopeptide antibiotic biosynthesis and resistance.^274,275^ A later study first used phylogenetic mining to identify relatives of GPA and then prioritized BGCs that lacked known resistance genes because these BGCs would be more likely to produce antibiotics with a novel mechanism of action (MOA). BGCs that appeared to have diverged from GPAs but lacked the GPA resistance genes were found to produce a known compound, complestatin, as well as a compound first identified in that study, corbomycin. Both compounds were found to be active against vancomycin-resistant and intermediate strains. These compounds work by binding to peptidoglycan and blocking autolysin activity, unlike “true-GPAs,” which target D-Ala-D-Ala peptidoglycan precursor, inhibiting transpeptidation/transglycosylation.^276^ These studies illustrate how phylogenetic mining can enable the discovery of structurally and evolutionarily related compounds with different MOAs and resistance profiles. Further study of synthetic or natural intermediates between the compounds could identify the structural motifs responsible for the divergent MOAs.

## 5. Machine learning analysis of biosynthetic gene clusters

**Fig. 9:**
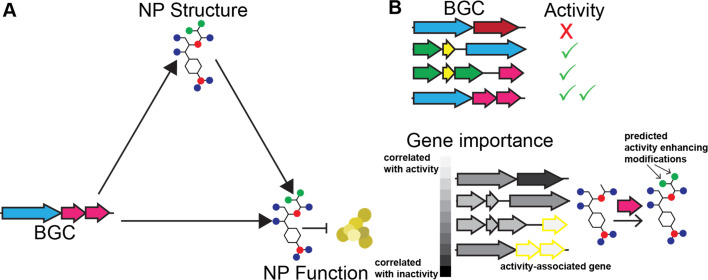
Use of BGC product activity prediction algorithms to infer NP SARs. (A) Relationship between BGC, NP structure, and NP activity. (B) Workflow for using methods that predict activity from BGCs to infer SARs.

As discussed above, variation in BGC genetic content leads to variation in product structure. With sufficient data and knowledge of biosynthetic enzymes, it should be possible to predict the structures of NPs from the sequence of the biosynthetic gene clusters that produce them. Similarly, with enough knowledge of SAR trends for the chemical scaffold of the product, it should be possible to predict activity from the structure of the product. Since BGC determines product structure and product structure determines activity, it follows that NP activity can be predicted directly from the sequence of the BGC encoding it (Fig. 9A). Beyond biosynthetic genes, BGCs also contain additional clues for the activity of their product since they often carry genes that provide resistance to the product. There are several methods for identifying these genes.^249,280^

Recently, there have been several machine learning methods reported that predict NP bioactivities from features of the BGC that produce them. While all of these methods have limited accuracy, likely due to a severe lack of training data, we expect that they will greatly improve in the future as more data and advanced AI models become available. SARs can be gleaned from these methods in two ways. First, explainable AI methods (discussed further below) can be used to identify which biosynthetic features contribute to a prediction of activity or inactivity (Fig. 9B). These biosynthetic genes can then be connected to the functional groups they install in the final product, which can also be assumed to contribute to activity or inactivity, respectively. Second, activity can be predicted for different natural variants of BGCs discovered using the methods described in the previous section. If the method is of sufficient accuracy to predict the relative activity of the products of the two BGCs and the structural change between the products can be determined from the BGCs, this could predict a SAR. While these methods may not currently be of sufficient accuracy for the second approach to work on most BGCs, we expect that in the future this type of analysis could become feasible. In the remainder of this section, we further describe how each of the reported prediction methods works and suggest how the method could be adapted for studying SARs of NPs.

The first reported method to predict NP activity from BGC was DeepBGC. DeepBGC's primary function is to identify BGCs using a deep learning approach, specifically a Bidirectional Long Short-Term Memory (BiLSTM) Recurrent Neural Network that takes a sequence of embedded protein domain family classifications (PFAM) vectors as inputs. For its activity prediction, DeepBGC uses a random forest model trained on a count vector of PFAM domains. Random forest models have a feature importance score that measures which features are most important for making classifications. If this approach is applied to DeepBGC, it could be used to identify biosynthetic genes which are correlated with activity and the structural motifs they install. DeepBGC is trained to predict four bioactivities: antibacterial, cytotoxic, inhibitor, and antifungal. The activity prediction of DeepBGC was only trained on 370 training data points and therefore has limited accuracy,^243^ and attribution of activity to specific biosynthetic domains using this model would also likely lack accuracy.

The next reported method to predict bioactivity from BGCs is PRISM 4. PRISM 4's primary function is to identify BGCs and predict the chemical structure of the product, but PRISM also has activity prediction functionality. The authors of the PRISM 4 study trained support vector machines (SVMs) to predict bioactivities and compared two different BGC featurization strategies – a PFAM count vector and the chemical fingerprint of the PRISM predicted product structures. They found that the models that used predicted structures were more accurate than those using PFAMs. PRISM 4 was trained to predict five bioactivities: antibacterial, antifungal, antiviral, antitumor, or immunomodulatory activity.^248^ In general, SVMs are less interpretable than the random forest method used by DeepBGC because SVMs often use non-linear kernel functions which mix features. Since the model is applied to predicted structures, it is possible to make changes to the predicted structure and to analyze how those changes impact the predicted probability of activity.

We previously reported a third method for predicting bioactivity from BGCs. This method relies on counts of not only PFAM domains but also other biosynthetic domain annotations supported by antiSMASH, predicted monomers for NRPS and PKS modules, and resistance genes annotated by the resistance gene identifier.^281^ We used three different models – random forest, logistic regression, and support vector machines in this study – and found that the identity of the model did not significantly impact accuracy. We trained the models to predict six activities: antibacterial, activity against Gram-positive bacteria, activity against Gram-negative bacteria, activity against eukaryotic cells, activity against fungus, and antitumor activity. As discussed above, random forests are interpretable due to their feature importance score as is the logistic regression which provides coefficients for each feature – with larger coefficients being more important for predictions. While it is possible to do this type of analysis using DeepBGC and PRISM 4's activity prediction methods, we were the first to report feature importance analysis for these types of predictions. Our models picked up on several known structure–activity trends, for example that amines are associated with activity against Gram-negative bacteria and that *N*-methylation of peptides is associated with activity against eukaryotic cells^282^ as well as some associations that have not previously been studied. Subsequently, our method was adapted for use on fungal BGCs as well as bacterial BGCs, although accuracy on fungal BGCs is currently hindered by a lack of training data.^283^

Each of the methods described above can predict bioactivity from the sequence of BGCs, either directly or by first predicting the structure of the product. Explainable AI tools, which will be discussed further in a later section, can then be used to reveal what biosynthetic or molecular features are correlated with activity. This process has been shown to reveal previously known SARs and predict additional SARs that have yet to be validated. Currently, these methods are severely limited by a lack of well-curated training data, which reduces their accuracy in activity prediction as well as in identification of SARs.

## 6. Structure based docking and modeling studies to predict SAR

### 6.1 Computational methods in drug discovery

**Fig. 10:**
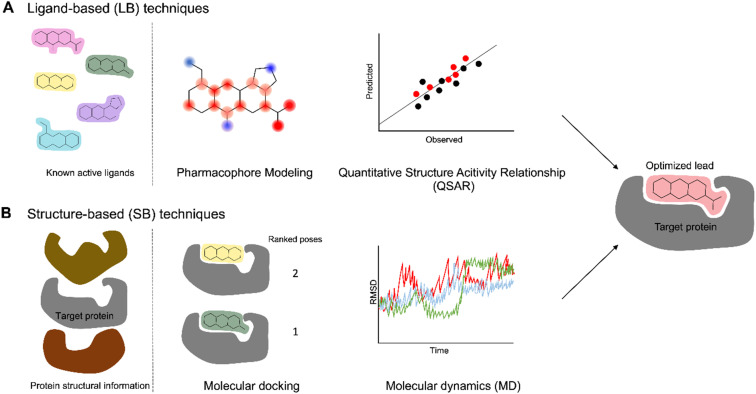
CADD strategies to study SAR. (A) Ligand-based methods primarily utilize information from known active molecules (B) structure-based techniques involve the 3D structures of target receptors.

While NPs provide us a gateway into their diverse structural and biological arsenal, the chemical space surrounding NPs is too vast to explore with experimental approaches alone. Improvements in technological resources, statistical methods, and structural biology advancements have propelled computational methods to the forefront as indispensable, time-efficient, and cost-effective tools in the field of drug discovery. These methods collectively fall under the umbrella term of computer-aided drug design (CADD) and are categorized into two general approaches: ligand-based (LB) and structure-based (SB) methods (Fig. 10). CADD methods have played a significant role since the 1960s^284^ and have been incorporated into every step of the drug discovery process from target identification to lead optimization. This approach has contributed to the development of various pharmaceuticals currently in clinical trials or approved for use including Captopril, Dorzolamide, Saquinavir, Zanamivir, Oseltamivir, Aliskiren, Boceprevir, Nolatrexed, Rupintrivir, Imatinib, Indinavir, Tirofiban, and Raltegravir.^285–288^ For more comprehensive information on these methodologies, additional details can be found in other reviews.^289–293^ Here, we briefly outline these methods and highlight their utility in exploring and predicting the SAR of analogs derived from NPs.

Ligand-based (LB) methods rely on the molecular similarity principle, where molecules with similar structural and physicochemical qualities are likely to share similar properties or activities (Fig. 10A). One such LB method is pharmacophore modeling which extracts essential molecular features in active ligands – such as electronegativity, symmetry, hydrogen bond donors and acceptors, aromaticity, and many more – to generate a model highlighting the common features among the ligands.^294,295^ Another widely used LB method is quantitative structure–activity relationship (QSAR) which elucidates significant and quantitative correlation between ligand properties, represented by 1D to nD numerical descriptors, and biological activity. Earlier works primarily relied on simple 1D and 2D descriptors such as molecular weight and logP while later works started incorporating higher dimensionality.^296^ QSAR models employ statistical techniques like multi-linear regression (MLR) and principal component analysis (PCA)^295,297,298^ while Comparative Molecular Field Analysis (CoMFA)^299^ and Comparative Molecular Similarity Indices Analysis (CoMSIA)^300^ have become prominent among the 3D-QSAR techniques.^301^ This can then be used to estimate the activities of related novel compounds based on their structural attributes. Like other LB methods, this approach is not explicitly dependent on the interaction of the molecule with its target protein. For example, to identify potential dengue protease inhibitors, LB-QSAR and pharmacophore models were developed from derivatives of 4-benzyloxyphenylglycine – an important residue in previously identified protease inhibitors.^302,303^ The models were used for virtual screening of similar features from ZINC database and resulted in identification of two promising compounds; subsequent docking studies validated their favorable binding with the dengue protease. Another study leveraged 2D and 3D-QSAR to design novel anti-osteosarcoma chemotherapy drugs. First, 2D-QSAR models were generated from dipeptide-alkylated nitrogen-mustard derivatives followed by construction of a CoMSIA model to account for the 3D spatial characteristics. Crucial descriptors identified from the 2D-QSAR experiments and the contour map from the 3D-QSAR model guided the design of 200 new nitrogen-mustard compounds which were screened against potential targets with docking.^304^ The LB approach enables the design of compounds even if the target is not known, but it requires proper identification and handling of molecular descriptors, adequate available data, and validation methods for high-quality LB models. Another potential limitation of LB QSAR models is that they rely on previously observed trends and are unlikely to correctly predict activity of compounds unrelated to those used to build the model.^290^

Structure-Based (SB) methods play an equivalently important role in drug design by leveraging the 3D structures of biologically relevant target proteins and elucidating their interaction with ligands. The two main SB techniques utilized are molecular docking and molecular dynamics (MD) simulations (Fig. 10B). Molecular docking is used to predict the preferred orientation and position of a ligand in the active site of a target protein, and scoring functions embedded in docking programs provide rapid and simplified quantitative assessment of the binding affinity and quality of ligand binding poses among the multiple conformations generated.^305,306^ These scoring functions, classified into physics-based, empirical-based, and knowledge-based, rely on atomic force-fields, physicochemical properties, and statistical analyses of protein-ligand complexes, respectively.^86,307^ The ability to rank ligand binding affinity *via* the scoring function facilitates the identification of modifications influencing binding strength, as illustrated by virtual screening studies applied to GPCRs.^308,309^ In another example, scoring functions were correlated with acetylcholinesterase (AChE) inhibition potency, showcasing a quantitative connection between scoring functions and activity.^310^ Meanwhile, a computational study on fatty acid binding proteins (FABP) guided the design of new class of antinociceptive and anti-inflammatory agents.^311^ SAR was established after docking studies, determining that the α-truxillic acid scaffold is essential for FABP binding, and identified two lead candidates after promising *in vivo* efficacy results. In these studies, reliable scoring functions were influential in distinguishing binders from nonbinders and in highlighting important molecular structures; however, the major weakness in most docking studies is the approximations used by the scoring functions, leading to low accuracy of the binding affinity.^305^ Docking can be further refined with techniques like free energy perturbation (FEP) and thermodynamic integration (TI) for improved binding free energy predictions, another indicator to characterize binding strength.^312–314^

While molecular docking may provide a static model of a protein–ligand interaction, it fails to accurately represent the inherent conformational flexibility exhibited by most biomolecules, limiting further meaningful SAR analysis. On the other hand, molecular dynamics (MD) simulations have the ability to probe the dynamic behavior of ligand–protein complexes over time and provide more accurate measurements of binding affinity. MD simulations help capture the flexibility and fluctuations in the complex structure using Newtonian mechanics. In the context of SAR studies, MD simulations are typically used to reevaluate the results of docking studies, providing additional quantitative insights into the strength and stability of the ligand–protein interaction. In order to obtain sufficiently comparable results to experiments, an equally important aspect in these simulations is that realistic solvent conditions are accounted for. Post-processing MD approaches like linear interaction energy (LIE)^315^ method and methods that utilize implicit solvent models such as Molecular Mechanics/Poisson-Boltzmann Surface Area (MM/PBSA) and Molecular Mechanics/Generalized Born Surface Area (MM/GBSA) are efficient in estimating binding free energies.^316–318^ The atomic detail obtained from MD simulations, especially for complex molecular interactions at longer time scales, are more computationally expensive than docking; nevertheless, this SB method provides a more robust calculation, serving as another metric for optimizing the pharmacological properties of drug candidates.

SB methods require knowledge of the target's structure, which were traditionally determined using spectroscopic techniques, including nuclear magnetic resonance (NMR), X-ray crystallography, cryo-electron microscopy, and homology modeling to provide reliable 3D structures of protein targets. Until recently, it was impossible to determine the structure of the vast majority of protein targets computationally, unless they had a close homolog that could be used as a template for modeling. More recently, AlphaFold has enabled the prediction of many protein structures, including those without any structurally characterized homologs.^319^ However, it is still unclear how suitable these models are for docking and other SB methods.^320–323^ Recently, several AI-based docking methods have been developed, which could have the potential to be faster and more accurate than traditional methods,^324–326^ but these methods generally do not perform well on benchmarks.^327^ This underscores the inherent and general limitations of computational methods due to complexity of biological molecules, availability and quality of data, and resource constraints. These computational methods essentially serve as approximations with varying levels of accuracy and experimental verifications are ultimately required to assess the impact of the results. However, comparisons to previously obtained experimental data are initially used to evaluate their performance. For SB-based methods, calculating the root-mean squared deviation (RMSD) of a docking pose or MD trajectory with respect to a structure from the aforementioned structural biology instrument is a common validation technique; satisfactory RMSD values are ≤2 Å. For LB-based methods, internal and external validation using datasets with experimental values and metrics like cross-validation are used. Despite these challenges, these calculations provide valuable insights, especially in SAR studies, into how variations in ligand structure influence binding affinity and binding free energies which translate to biological activity. Additionally, they enable extremely high throughput studies that are not possible to accomplish in the wet lab.

### 6.2 Applications of CADD to natural product SAR

Most examples of CADD have used primarily unnatural compounds. But, CADD technology is just as applicable to NPs as it is to synthetic compounds, although conformational search for NPs will often be slightly more challenging due to their general higher complexity and number of rotatable bonds. The chemical space surrounding a known NP, or general areas of NP-like chemical space (*e.g.* peptides made up of amino acids found in NRPS or RiPPs) can be used to create a library for virtual screening. Virtual screening is the process by which docking and other CADD techniques are applied to large libraries of chemical structures.^328^ SARs can be derived from the results of the virtual screen and confirmed with additional targeted experiments designed based on the results of the virtual screen. It is generally possible to screen many more compounds by virtual screening than by experimental screening. This is especially true for NPs, where their analogs must first be obtained by synthesis, biosynthesis, or isolation from a natural source, all of which are costly. Therefore, we propose that virtual screening should be incorporated into NP drug discovery efforts more than they currently are.

Despite the focus on unnatural compounds in most virtual screens, there have been a few studies that applied CADD methods to NP drug discovery. Sometimes, these efforts focus on optimizing a single NP scaffold. For example, the Shenvi group used docking to determine if a proposed stable analog of Salvinorin A was still able to bind the κ-opioid receptor before investing in the synthesis of the analog.^29^ Conversely, complexes predicted by docking can be used to rationalize experimentally observed differences in binding affinity, as was done in a study of synthetic cannabidiol analogs with activity against the μ-opioid receptor.^329^

Other studies have performed virtual screening using large libraries of NP. Available libraries include databases that contain NP structural data such as NPAtlas,^263^ COCONUT,^330^ Canvass,^331^ and the ZINC library.^332^ The ZINC library contains both synthetic and natural compounds, but it is especially useful in screening since many of the compounds in the library can be purchased, enabling easy experimental follow up experiments for any virtual hits. Ideally, multiple techniques described in the previous section can be combined to improve the efficiency and accuracy of the virtual screen. There are several examples for studies that combined pharmacophore-based and molecular docking screening applied to NP libraries against the following targets: X-linked inhibitor of apoptosis protein,^333^ the SARS-CoV2 Main protease,^334^ and enzyme 5-enolpyruvylshikimate-3-phosphate synthase.^335^ Other studies have used a combination of docking and MD to screen for inhibitors of the following targets: penicillin binding proteins and β-lactamases,^336^ Fascin,^337^ the SARS-CoV2 Main protease,^338^ and RAF and MEK kinases.^339^ One limitation of these studies is that there are not many NPs that are commercially available, so it is difficult to experimentally validate any hits. One study addressed this challenge by using extracts from herbs that were more likely to be rich in the hit from the virtual screen.^340^ While CADD is still limited by a lack of accuracy, we believe that it is still a useful tool, especially when combined with creative computational-experimental feedback loops and therefore we expect it to play an increasingly important role in NP drug discovery in the future.

## 7. Explainable AI/ML models for analysis of SAR

### 7.1 Overview of AI/ML and SAR in small molecules

**Fig. 11:**
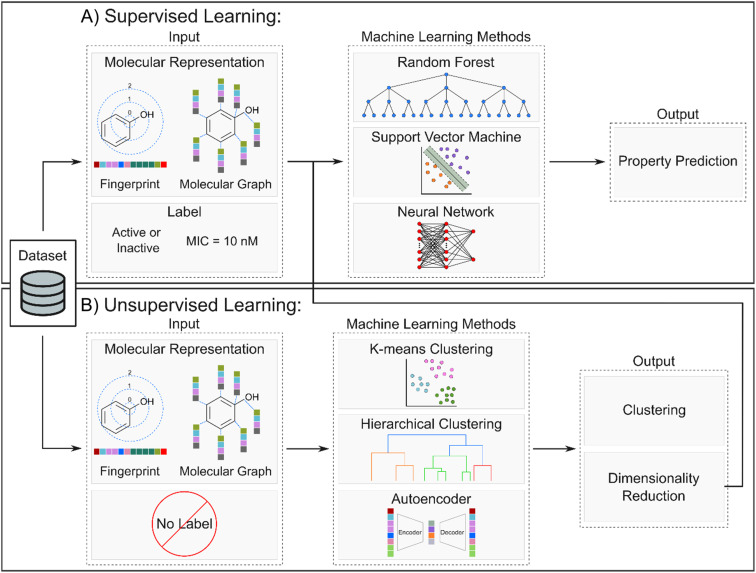
Overview of ML workflows used for the SAR analysis of small molecules. The workflows are split into two categories: (A) supervised learning and unsupervised learning. In supervised SAR models, ML is utilized for property prediction. (B) In unsupervised SAR models, the ML methods are mainly used for clustering or dimensionality reduction.

In the past few decades, machine learning (ML) has been increasingly utilized in the SAR field to develop ML-based SAR models. ML is a subfield of artificial intelligence (AI) that uses data and algorithms to identify patterns and make predictions. The integration of ML has allowed for more complex, nonlinear approaches to SAR analysis.^341^ ML can be broken down into two categories, supervised or unsupervised learning (Fig. 11). This review will mainly focus on supervised learning (Fig. 11A) which uses data labeled with a prediction or classification. In ML-based SAR models, supervised learning is utilized to predict properties of compounds like bioactivity or ADMET (absorption, distribution, metabolism, excretion, and toxicity).^342,343^ Unsupervised learning uses unlabeled training data and identifies patterns without any guidance or human oversight. It is useful in ML-based SAR models to learn general patterns of chemical structures to generate feature representations of the data^344,345^ or cluster similar compounds together.^346^ In addition to drug discovery, ML-based SAR models have also been applied in materials^347^ and organic synthesis.^64,348^ This review section will be focused on the usage of ML to predict biological activity, and its potential applications to the study of NP SAR.

To predict SAR with ML techniques, curated molecular datasets must first be encoded into numerical representations. The encoded compounds, termed molecular representations or molecular descriptors, can be represented in 1D, 2D, 3D, or even higher dimensions.^349^ The most common representations are the 2D-molecular descriptors which include information on the atoms and their connectivity. Popular 2D-molecular representations are the molecular fingerprints and the molecular graph.^350^ ML algorithms then use these molecular descriptors to find relationships between the molecular structure and the property of interest. ML algorithms range from interpretable linear models, such as linear regression, to more complex deep neural networks (DNNs). Although the more complex models have shown higher prediction accuracy, they do so at the expense of the interpretability of the model.^351–353^ Common ML models used in SAR analysis, such as random forest (RF), support vector machines (SVMs),^354^ and DNNs,^355^ are termed “black-boxes” as they lack the interpretability of linear models. In other words, users are unable to inherently understand how black-box models make their predictions. To address this, the field of explainable artificial intelligence (XAI) has emerged to develop methods to interpret black-box models.

### 7.2 Explainable artificial intelligence

XAI is a broad concept, and in this section, we aim to define the most commonly used terminology and why XAI is needed in ML-based SAR models. The definitions of two terms, explainability and interpretability, have been under debate in literature as some researchers use them interchangeably and others define them as separate concepts.^356,357^ In this review, explainability and interpretability will be defined separately. Explainability is an active characteristic of a model, providing an explanation of its decisions by using separate algorithms to understand its internal functions or logic.^356,358^ On the other hand, interpretability is defined as a passive characteristic and refers to a model that a user can inherently understand.^359^ Under these definitions, linear models and decision tree models are interpretable, whereas black-box models are not.

XAI is a useful technique for ML-based SAR models. Knowledge of what portions of the chemical structure the model deems to be an important predictor of bioactivity adds additional support to any predictions the model makes. This helps avoid the Clever Hans effect, which occurs when a model learns spurious correlations in the data, *i.e.*, the model produces correct predictions for the wrong reasons.^360^ It also helps bridge the gap between the scientific and machine learning communities as XAI provides justifications to predictions that could affect humans and has the potential to improve human understanding of SARs.

### 7.3 Types of XAI

**Fig. 12:**
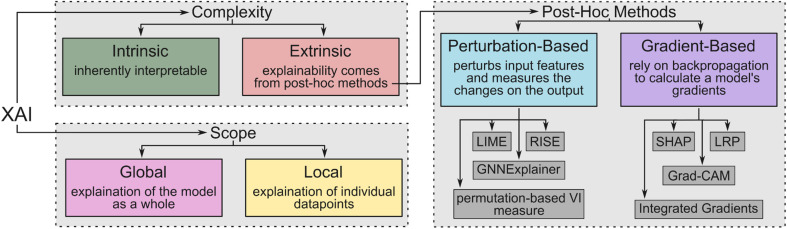
Taxonomy scheme of XAI methods. XAI models can be classified by their complexity and scope. Those classified as extrinsic require *post hoc* methods for explainability.

XAI has been categorized in multiple ways. In this review, we will classify the types of XAI methods based on a taxonomy scheme (Fig. 12) in a previously published survey which is based on complexity, level of dependency, and scope.^361^ The complexity of the model often determines how dependent the XAI technique is on the model, so these classifications will be grouped together.

XAI models classified by their complexity are either intrinsic or extrinsic. For intrinsic models, explainability comes directly from an interpretable model. Intrinsic XAI methods are model-dependent, meaning they can only be applied to specific models, and include simple, white-box models like linear or decision-tree models. For extrinsic models, explainability comes from *post hoc* methods which are applied to the model after training. Explainable methods for deep learning models fall under the category of extrinsic models as they require separate *post hoc* methods to understand their decisions. Many *post hoc* techniques are model-agnostic, meaning they can be applied to any model.

The scope of an XAI model refers to whether explanations look to understand the model as a whole (global interpretations) or understand individual datapoints (local interpretations). In ML-based SAR models, global interpretations capture general SAR trends and would typically contain multiple SARs. Global interpretations are useful when using a structurally and chemically diverse dataset. Conversely, local interpretations capture SAR trends of individual compounds, identifying functional groups or structural motifs that affect bioactivity. Local interpretations are useful in the optimization stage of drug development when researchers look to improve bioactivity and/or the ADMET profile.^353^

### 7.4 Common XAI methods in SAR of small molecules

SAR models of small molecules are typically interpreted by determining the descriptor importance which identifies correlations between descriptors and the predicted property.^351,362^ If the molecular descriptors are fingerprint- or graph-based, visual explanations can be created that highlight substructures identified as important in predicting the property. Visual explanations for small molecules include colored molecules and heat maps that color atoms or bonds based on their importance.^363,364^ This importance can be based on models trained on activity without target structural information (ligand based approach) or on protein–ligand structures labeled with binding affinity, in which case the importance should approximate contribution of a group to ligand binding affinity.^365^ It should be noted that the selection of molecular descriptors when developing ML-based SAR models is important and can affect the explainability of the model. Interpretable descriptors are those that have clear physio-chemical meaning and include various 1D descriptors (*e.g.*, molecular weight, the number of hydrogen donors, *etc.*) and topological descriptors. This section is not intended to serve as an exhaustive review of all XAI techniques, but rather to highlight XAI methods that are useful in the SAR analysis of small molecules and readers should refer to existing reviews for more details.^356,358,366^

Feature attribution techniques are *post hoc* methods that calculate an attribution score for each feature based on their contribution to the model's prediction. Feature attribution methods can be split into two broad categories: perturbation-based and gradient-based. Perturbation-based methods mask or modify each input feature to measure their effect on the output of the model.^367^ These methods are typically model-agnostic as they do not need access to the inner workings of a model. However, they require multiple passes through the network to calculate feature importance and as such are less computationally efficient than gradient-based techniques. Examples of perturbation-based methods include Local Interpretable Model-Agnostic Explanations (LIME),^368^ the permutation-based variable importance (VI) measure,^369^ Randomized Input Sampling for Explanation (RISE),^370^ and GNNExplainer.^371^

LIME is a local model-agnostic method that explains a model's predictions through a surrogate model^368^ by perturbing the input features for a specific instance and then observing the model's corresponding predictions. These results are then used to train a simple interpretable model (*e.g.*, linear model or decision tree) to approximate the original model's behavior in proximity to a specific instance. Whitmore *et al.* used LIME to provide structural interpretation for a model trained to predict research octane number.^372^ A large problem of LIME is its sampling technique, which can lead to unlikely data^373^ and frequent generation of unstable explanations for complex, nonlinear models.^374^ In other words, for complex models, LIME can generate very different explanations for neighboring inputs that have only been slightly modified.

The permutation-based variable importance (VI) measure was first proposed by Breiman for random forest models.^369^ A model-agnostic version called model reliance has since been adapted by Fisher *et al.*^375^ This technique measures the change in the prediction error after permuting the input features. Important features cause a large increase in error after permutation. Guha and Jurs developed a variant of this method for CNN SAR models.^376^

RISE, which is generally applied to tasks with image input data, estimates feature importance by multiplying each input elementwise with random masks and measuring the model's response.^370^ From this, the method generates saliency maps from linear combinations of the masks. To our knowledge, RISE has yet to be used to explain a ML-based SAR model. However, it has been used to generate instance level and model level explanations for a pollen classification model trained on fluorescence spectra and shows promise for explaining small molecule image data.^377^

**Fig. 13:**
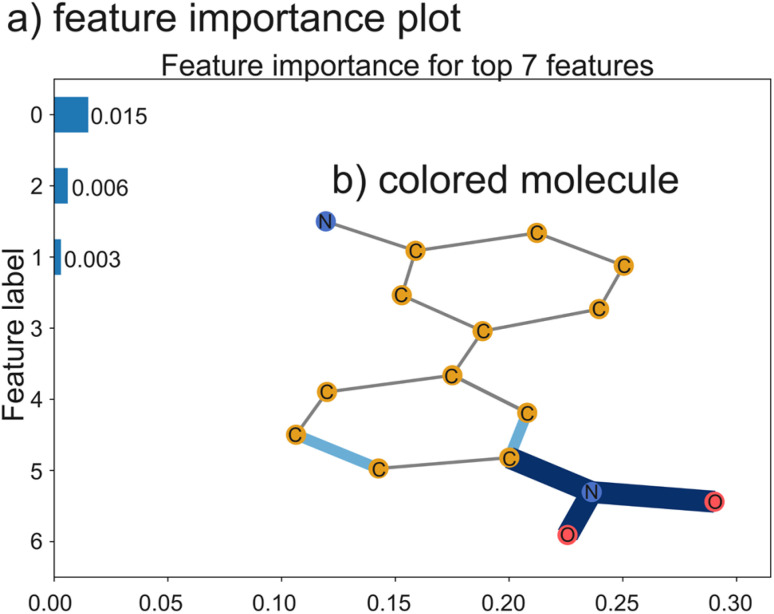
Generated explanation of a SAR model using GNNExplainer.^371^ A GNN was trained on the MUTAG dataset which contains the mutagenetic data of nitroaromatic compounds. The subsequent predictions were explained with GNNExplainer. The methods to do this were based off of the blog post Why should I trust my Graph Neural Network? and its associated colab.^379^ (a) A feature importance plot generated by GNNExplainer for the compound. (b) Visualization of the explanation for this compound. Edges (the bonds) colored blue indicate high mask areas that the model deemed important for the prediction task. The darker the blue, the more important the bond was. For this molecule, NO_2_, a known mutagenetic substructure,^380^ was highlighted as important when predicting the molecule as mutagenetic.

GNNExplainer is applicable to any graph neural network (GNN)-based model.^371^ It provides explanations of a GNN's predictions by learning a graph mask and a feature mask that mask unimportant features of the input. To do this, GNNExplainer randomly initializes the masks and then optimizes them by maximizing the mutual information between the predictions of the original graph and the perturbed graph. By learning the unimportant features of the input graph, GNNExplainer can provide the important subgraph and node features that affect the model's predictions (Fig. 13). Wojtuch *et al.* recently used this technique to determine important molecular features of models trained on four datasets: the ESOL dataset (a water solubility dataset), the QM9 dataset (a quantum properties dataset), a human metabolic stability dataset, and a rat metabolic stability dataset.^378^

Gradient-based methods rely on backpropagation to compute the gradients of the model's output with respect to each input feature,^381^ which are then used to estimate attribution scores. Gradient-based methods are model-dependent as they can only be used on models trained by gradient descent. They also tend to be noisy, producing feature importance maps with irrelevant contributions.^382^ Examples of gradient-based techniques used in ML-based SAR models are gradient-weighted class activation maps (Grad-CAM),^383^ Integrated gradients,^384^ Layer-wise Relevance Propagation (LRP)^385^ and Shapley Additive Explanations (SHAP).^386^

Grad-CAM is a flexible version of class activation maps (CAM) that can be used on any convolutional neural network (CNN) architecture. Grad-CAM utilizes the gradients in the final convolutional layer of CNNs to visualize the regions of the image the CNN used for classification.^383^ It has been used to interpret many small molecule SAR models, including by Zhong *et al.* to interpret SARs for predicting the rate constant of a compound's reaction with OH radicals and validate the model by visualizing regions that were linked to the model's predictions.^387^

Integrated gradients is another popular gradient-based technique that was designed to satisfy what Sundararajan *et al.* describe as two fundamental axioms of attribution methods: sensitivity and implementation invariance. To determine the important features of a deep neural network, integrated gradients compute the average of all gradients along a path from a baseline input (defined as an input where the prediction is neutral or near zero) to the actual input.^384^ Integrated gradients have been utilized to investigate protein–ligand binding, cytochrome P450 inhibition, hERG channel inhibition, and passive permeability.^388,389^ This technique was able to discern known important molecular features of these properties as well as identifying models that achieved high prediction accuracy by learning spurious correlations.

LRP interprets predictions of black-box models through backpropagation.^385^ It begins with the output layer of the model, assigning relevance to each neuron. The relevance is then backpropagated through the network to the input-layer neurons using a set of designed local propagation rules. LRP is not inherently gradient-based. However, a variant of LRP, ∈-LRP, can compute the average gradient and as such, LRP is typically classified as a gradient-based technique.^381^ An example of the use of LRP in ML-based SAR models includes Baldassarre and Azizpour's usage of LRP to explain a graph neural network trained to predict the aqueous solubility of organic compounds.^390^

SHAP is a technique that combines three linear explanation models – LIME, LRP, and DeepLift – with three classic Shapley value estimations.^386^ Shapley values are derived from cooperative game theory and were originally used in economics to fairly distribute resources within a group (such as dividing profits or payouts) by determining each player's contribution to the game. Lundberg *et al.* developed both model-agnostic and model-specific approximation techniques for calculating Shapley values to explain ML models.^386^ For example, Kernel SHAP, a model-agnostic technique, combines Linear LIME and Shapley values, whereas Deep SHAP, a model-specific technique, combines DeepLIFT and Shapley values. This method satisfies three desirable properties of additive feature attributions: local accuracy, missingness, and consistency. In small molecule SAR analysis, SHAP has been used to determine compound substructure features that affect metabolic stability^391^ and bioactivity.^392^

### 7.5 Applications of XAI in SAR of NP

ML-based SAR models of NPs have only recently begun to grow in popularity. This is due, in part, to the fact that curated and freely available NP databases of sufficient size and quality for ML have only recently become available. Considering the abundance of NP or NP-derived drugs,^2^ SAR models of NPs are commonly developed to predict bioactivities. Some commonly predicted bioactivities include anti-cancer,^393–396^ anti-microbial,^397–400^ and anti-inflammation.^401,402^

Popular encyclopedic NPs databases include NPAtlas,^263^ COCONUT,^330^ and the Universal Natural Product Database.^403^ Most notable is COCONUT, which is a large database containing the largest and most diverse collections of NPs. Many other databases only contain a particular type of NP, like NPAtlas which focuses on microbial NPs, while others are no longer updated or supported. These encyclopedic databases mainly contain structural information and do not contain information on bioactivities. To train a ML model to predict bioactivities, more specialized databases are needed. For example, anti-cancer NPs can be found in the NPACT^404^ or NPCARE^405^ databases. Sorokina and Steinbeck's review gives a more in-depth survey of the current state of NP databases.^406^

**Fig. 14:**
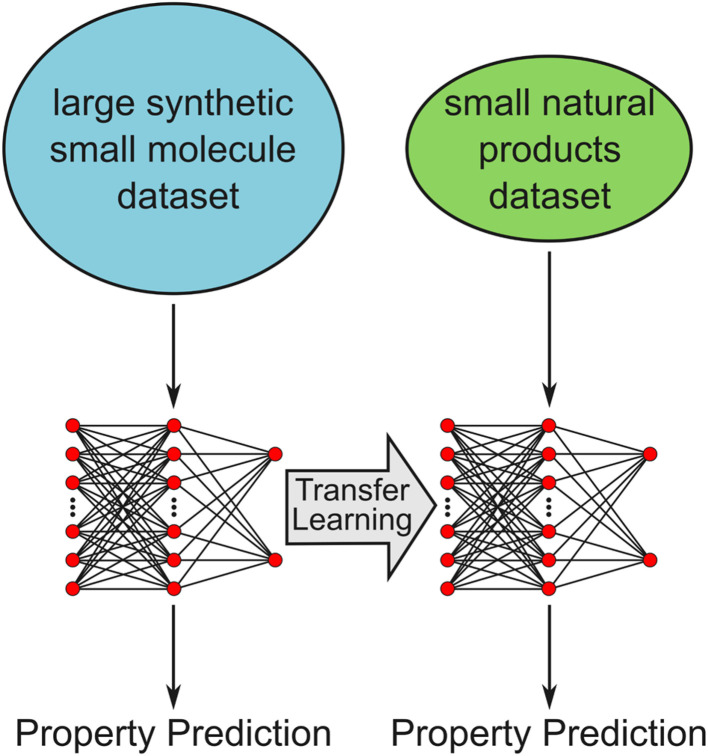
Overview scheme of transfer learning in ML-based SAR models of NP. An ML model trained on a large dataset of synthetic small molecules can be fine-tuned on a smaller NPs dataset for the property prediction of NPs.

Despite the growing number of databases in the field, there is still a lack of publicly available NPs bioactivity information. For this reason, many ML-based SAR models of NPs are trained on datasets containing synthetic small molecules. However, given the difference between small molecules and NPs (NPs typically have greater molecular weights, more hydrogen bond donors/acceptors, more oxygen atoms, fewer nitrogen atoms, *etc.*), ML-based SAR models of small molecules are not inherently translatable to NPs as NPs are outside of these models' applicability domains,^407^ or region in chemical space, defined by the model's training set, for which the model can make reliable and accurate predictions. One potential solution is transfer learning, a type of ML that is used when there is not sufficient training data for the task of interest. The learned parameters of a model pre-trained on one task, like the bioactivity of small molecules, can be transferred or fine-tuned to a model for a new task or domain, like the bioactivity of NPs (Fig. 14).^408^ Qiang *et al.* used this technique to fine-tune a model pretrained on ChEMBL data to predict multiple targets for NPs.^409^

However, the use of XAI in ML-based SAR models of NPs is still lacking. The most common XAI application in the area is in the classification of compounds as NPs. Kim *et al.* used a supervised feed-forward network to classify the structure of a NP into three levels: pathway (specialized metabolism), superclass (taxonomic information and chemical properties), and class (chemical structure).^410^ Although the authors did not use any of the XAI techniques described in this review section, they did manually study the response of NPClassifier to perturbations in NP input structures to determine what structural features the model was using and why the model misclassified structures. NP-Scout, developed by Chen *et al.*, is another ML method to classify small molecules as NPs.^411^ The classified molecules were visualized using similarity maps^412^ to highlight portions of the molecule that the random forest model used to classify as either a NP or a small molecule. To our knowledge, the only instance of one of the previously described XAI techniques being used in a ML-based SAR model of NPs was from Maroni *et al.*^413^ This model was trained on both natural and synthetic molecules to classify compounds as either sweet or bitter. They used SHAP to obtain global explanations and local explanations of the model's decisions.

As the use of black-box models in the SAR analysis of NPs continues to rise, so should the subsequent use of XAI techniques. Any of the XAI methods described in this review can be utilized in ML-based SAR models of NPs. Considering the many applications of NPs in the drug discovery field, XAI can foster collaboration between the scientific and machine learning community by providing explanations to predictions. In addition to giving insight into the model's decisions, any identified substructures or features could guide optimization of lead compounds. Going forward, we recommend that any results from a ML-based SAR model of NPs be backed by explanations from an XAI technique.

## 8. Conclusion

In this review, we have presented experimental and computational methods that can be used to study the SARs of NPs. All of these methods are complementary. Different approaches to NP synthesis, derivatization, biosynthesis, and isolation are likely to give access to different analogs. We have presented several examples, such as the antibiotics daptomycin, which have been studied using multiple of these techniques, illustrating their complementarity. However, many computational techniques, in particular QSAR models and XAI models, require experimental data to build the models. Therefore, we propose that the optimal way to study NP SAR is through an experimental–computational feedback loop in which experiments are used to validate and generate training data for computational studies and computational studies are used to focus synthetic and biosynthetic efforts on those compounds that are most likely to have improved activity or be informative for computational model refinement. Successful execution of such a feedback loop requires expertise in many domains ranging from chemical synthesis, bioactivity assay development, synthetic biology, bioinformatics, cheminformatics, and artificial intelligence and will therefore likely require collaboration between researchers in the NP field. We expect that these collaborative efforts will play a key role in drug development in the future, especially for emerging threats such as antimicrobial resistant pathogens and future pandemics.

## 9. Conflicts of interest

There are no conflicts to declare.
